# Chemical Profile and Biological Effects of an Herbal Mixture for the Development of an Oil-in-Water Cream

**DOI:** 10.3390/plants12020248

**Published:** 2023-01-05

**Authors:** Diana Antonia Safta, Irina Ielciu, Raffaela Șuștic, Daniela Hanganu, Mihaela Niculae, Mihai Cenariu, Emoke Pall, Mirela Liliana Moldovan, Marcela Achim, Cătălina Bogdan, Ioan Tomuță

**Affiliations:** 1 Department of Dermopharmacy and Cosmetics, Faculty of Pharmacy, “Iuliu Haţieganu” University of Medicine and Pharmacy Cluj-Napoca, 400010 Cluj-Napoca, Romania; 2 Department of Pharmaceutical Botany, Faculty of Pharmacy, “Iuliu Haţieganu” University of Medicine and Pharmacy Cluj-Napoca, 400010 Cluj-Napoca, Romania; 3 Department of Pharmaceutical Technology and Biopharmacy, Faculty of Pharmacy, “Iuliu Haţieganu” University of Medicine and Pharmacy Cluj-Napoca, 400010 Cluj-Napoca, Romania; 4 Department of Pharmacognosy, Faculty of Pharmacy, “Iuliu Haţieganu” University of Medicine and Pharmacy Cluj-Napoca, 400010 Cluj-Napoca, Romania; 5 Department of Clinical Sciences, University of Agricultural Sciences and Veterinary Medicine Cluj-Napoca, 400374 Cluj-Napoca, Romania

**Keywords:** QbD approach, cosmetic cream, seborrheic dermatitis, *Hamamelis virginiana* leaves, *Krameria lappacea* root, *Salix alba* bark

## Abstract

Three individual hydroalcoholic extracts derived from *Hamamelis virginiana* leaves, *Krameria lappacea* root, *Salix alba* bark, and the resulting herbal mixture (HM) were assessed for the phytochemical profile as well as for antibacterial and cytotoxic potential. The chemical composition of the individual extracts and of their mixture was analyzed by chromatographical (LC-MS) and spectrophotometrical methods. The antimicrobial properties were evaluated by using the agar-well diffusion and the broth microdilution assays, whereas the potential cytotoxicity was investigated on human keratinocyte cell line by MTT method and apoptosis test. The HM composition revealed important amounts of valuable polyphenolic compounds provided from the individual extracts, having synergistic biological effects. All tested extracts displayed in vitro antimicrobial properties, with a significantly higher efficacy noticed for the HM when tested against *Staphylococcus aureus*. Moreover, none of the tested extracts was responsible for in vitro cytotoxicity against the human keratinocytes in the selected concentration range. Furthermore, the HM was included in an oil-in-water cream for the nonpharmacological treatment of seborrheic dermatitis, developed and optimized by using a QbD approach. A D-optimal experimental plan with four factors that varied on two levels was used to investigate the effect of the quantitative variation of the formulation factors (emulsifier, co-emulsifier, thickening agent, oily phase ratio) on the characteristics of the cream in terms of firmness, consistency, adhesiveness, stringiness, spreadability, and viscosity. Based on the experimental results, an optimal formulation containing 2.5% emulsifier and 20% oily phase was prepared and analyzed. The obtained results showed appropriate quality characteristics of this novel cream, which may be used in the future to manage the associated symptoms of seborrheic dermatitis.

## 1. Introduction

Seborrheic dermatitis (SD) is a common inflammatory skin disease that is most frequently found in the infancy and middle-aged population, with a papulosquamous morphology in areas rich in sebaceous glands, especially the scalp, face, and body folds [1,2]. The prevalence of SD worldwide is approximately 5%, especially affecting young adults or adolescents [1]. Its pathogenesis is multifactorial, and factors such as cutaneous microbiome alterations, excessive sebaceous gland activity, or immunosuppression appear to be involved in it [2,3]. Among microbiome alterations, the *Malassezia* yeast is often assumed to cause SD [3,4]. However, the bacterial flora of the skin may also be involved in the pathogenesis of SD, the most common bacterial agent being *Staphylococcus aureus* [4].

The management of SD involves the use of several therapeutic approaches, including topical corticosteroids and antifungals [1,2]. Due to their side effects and low tolerance, many patients search for safer alternatives, which often are natural remedies of different types [5,6,7]. Herbal extracts have traditionally been used for centuries for their important role in maintaining and enhancing the appearance of the skin. Herb-based cosmetics are perceived by consumers as safe and effective; consecutively, in recent years, the demand for natural and sustainable ingredients is continuously increasing [6,7]. *Hamamelis virginiana* L., *Salix alba* L., and *Krameria lappacea* (Dombey) Burdet and B.B. Simpson represent some of the most valuable vegetal species with cosmetic properties.

*Hamamelis virginiana* L. (witch-hazel, Hamamelidaceae) leaves have powerful antioxidant and astringent properties. The main constituents of the extract include tannins (e.g., condensed tannins—gallocatechin, epigallocatechin, catechin, epicatechin and hydrolysable tannins—hamamelitanin), gallic acid, catechins, proanthocyanins, flavonoids (kaempferol, quercetin), volatile oil (rich in carvacrol, eugenol), and triterpenic saponins [8]. The medicinal use of *H. virginiana* leaves in minor skin lesions, bruises, dermatitis, and sensitive scalp is supported by clinical data [9].

The phytochemical composition of the *Salix alba* L. bark (willow bark, Salicaceae), includes phenolic compounds (e.g., salicylic glycosides—salicin, salicortin) and tannins, the main component being salicin, an analogous precursor of the acetylsalicylic acid. These compounds are mainly responsible for their antiinflammatory, analgesic, and astringent properties [10].

*Krameria lappacea* (Dombey) Burdet and B.B. Simpson (syn. *Krameria triandra* Ruiz & Pavon) (krameria, Krameriaceae) roots contain the main metabolites represented by tannins, lignans (e.g., rataniaphenols) and proanthocyanidins, which support the antimicrobial, antioxidant, and astringent properties [11].

Despite their extensive use in the cosmetic industry, naturally derived ingredients may exhibit significant variations in terms of composition, rheological properties and stability of the final product [12]. Thereby, new approaches such as quality by design (QbD), which is a systematic, scientific, risk-based, and proactive method for developing and optimizing products or processes, emerge as an innovative approach to cosmetic development. The use of Design of Experiments (DoE), a tool of QbD, grants the acquisition of high-quality cosmetic products with desired characteristics. It helps to develop quality products by achieving a high degree of robustness. Moreover, it offers several advantages over traditional methods, such as a minimal number of experimental runs and reduced consumption of raw materials [13,14].

The purpose of the present study was to develop an optimized oil-in-water cream for the nonpharmaceutical treatment of facial SD. Taken into consideration the medicinal potential of the *K. lappacea, H. virginiana,* and *S. alba* species for overcoming the most important symptoms in SD, a mixture of the three herbal extracts (HM) was incorporated into the formulation. In fact, the choice of the three species was made considering the most important symptoms to be improved in SD. This mixture of extracts provides important chemical components for the treatment and relief of the SD symptoms such as tannin-type polyphenols (from *K. lappacea* and *H. virginiana*), flavonoids (from all three species) with antibacterial, astringentand antiinflammatory effects, as well as phenolic acids, including salicylic acid with exfoliating properties that can remove dead cells of the epidermis. The beneficial antimicrobial effects of the HM, in the context of the lack of cytotoxicty are supported by polyphenolic compounds, as multifunctional, plant-extracted bioactive compounds. Polyphenolic compounds are composed of tannins, flavonoids, and phenolic acids [15]. Their strong antioxidant and antiinflammatory effects and other beneficial properties for damaged skin can recommend polyphenols as a promising approach for the natural treatment of SD [16]. In addition to the herbal extracts, several active ingredients, such as allantoin, urea, glycerine, avocado oil, almond oil, and argan oil were added, due to their favourable properties for the fragilized skin. The novelty of this study is represented by the combination of extracts proposed for their potential synergistic and complementary effects in SD-related skin changes and by the experimental approach to developing the herbal-based cream. Even though the QbD approach has been well-documented in drug development over the past few decades, its usage in cosmetics development is still in its infancy, making this work noteworthy. The chemical composition of the HM and individual extracts was evaluated by using spectrophotometric and LC/MS methods, and cytotoxicity and antibacterial activity were also assessed for all these samples to sustain the inclusion of the HM in the proposed formulation. The HM was therefore incorporated in an oil-in-water cream. A D-optimal experimental plan with 19 formulations was used to investigate and optimize the influence of the quantitative variation of the formulation factors (the emulsifier, co-emulsifier, thickener, and oily phase ratio) on the critical physical attributes of the oil-in-water creams. In this way, an optimal and, at the same time, an innovative formulation was obtained, having significant biological activities and appropriate texture properties, which may be recommended for the control of seborrheic dermatitis.

## 2. Results and Discussions

### 2.1. Phytochemical Analysis of the Individual Extracts and of the HM

The HM included in the formulation was prepared as a combination in equal parts of three individual hydroalcoholic extracts from *K. lappacea* roots, *H. virginiana* leaves, and *S. alba* bark. The determined spectrophotometrical total polyphenolic content was 8.41 ± 0.051 mg GAE/mL for the HM, 8.376 ± 0.015 mg GAE/mL for the *S. alba* extract, 7.096 ± 0.028 mg GAE/mL for the *K. lappacea* extract, and 9.976 ± 0.052 mg GAE/mL for the *H. virginiana* extract. Individual polyphenolic components (Table 1) were identified and quantified by using a LC/MS method.

Concerning the individual extracts, some specific components, such as ferulic and salycilic acids were only identified for the *S. alba* extract, whereas trans-*p*-coumaric acid, catechin, and quercitrin were identified only for *H. virginiana* extract. The common components of the three individual extracts assure the enrichment of the HM in these compounds. The obtained results for the HM show significant amounts of flavonoids (rutoside, quercetin, myricetin, hyperoside, naringenin), but also of phenolic acids (carnosic, gallic, caffeic, chlorogenic, and salycilic acids). Being the most quantitatively important polyphenolic compounds, chlorogenic acid, salicylic acid, phenolic acids, and naringenin (as a flavonoidic compound) were obtained.

Some of these polyphenols were previously quantified in the composition of the individual species: e.g., flavonoids such as quercetin and its derivatives and phenolic acids such as caffeic acid and its derivatives in the composition of *S. alba* [17]. Catechin and its derivatives were previously reported for the composition of *K. lappacea* [18], whereas for *H. virginiana,* the tannins were found to be the main compounds [19]. However, the results for quantification of compounds are scarce for the three species, which make the results of the present study become important, especially as it is the first report that quantifies their mixture. Therefore, the present study represents the first report that quantifies all these individual compounds in the composition of the mixture. Moreover, to the best of our knowledge, naringenin was not previously reported in the composition of any of the three species, this being the first such report in scientific literature.

### 2.2. Assessment of the Antibacterial Activity of the Individual Extracts and of the HM

Results of the in vitro antimicrobial potential screening of the individual extracts and of the HM are presented in Table 2 and Table 3.

The tested individual extracts exhibited in vitro antimicrobial activity against all selected bacterial reference strains, except for *Pseudomonas aeruginosa*. Overall, both the individual extracts and the herbal mixture were able to inhibit bacterial growth, whereas their in vitro efficacy varied depending on the bacterial strains and the herbal species. Considering the values obtained for the diameter of the inhibition zone (Table 2), the highest potential was expressed against the Gram-positive species (MSSA > *Bacillus cereus* > *Enterococcus faecalis* > MRSA), compared to the selected Gram-negative (*Escherichia coli* and *Salmonella* Enteritidis). The most intense inhibitory activity was recorded in case of HM, with significantly higher diameter zones (*p* < 0.05) compared to those determined by individual extracts. When tested against MSSA, its efficacy was higher than that of individual extracts, similar to that of two of the positive controls (gentamicin and/or amikacin (*p* > 0.05)), but significantly lower (*p* < 0.05) compared to amoxicillin -clavulanic acid. However, when evaluated against the Gram-negative bacteria, HM presented a significantly weaker efficacy compared to all positive controls (*p* < 0.05) As a particular aspect, the antimicrobial activity was also recorded against the *Enterococcus faecalis* strain.

These results were in accordance with those obtained for the minimum inhibitory and bactericidal concentrations (Table 3) indicating a higher antimicrobial ability toward the Gram-positive strains. Indeed, the lowest values established by using the broth microdilution method were noticed when testing the product against *Staphylococcus* strains (MSSA and MRSA), *Bacillus cereus,* and *Enterococcus faecalis* (Table 3). Considering the MIC index, the tested extracts manifested bactericidal activity toward all tested bacterial species (MBC/MIC ≤ 4).

The in vitro antimicrobial efficacy of the HM against Gram-positive strains was found similar to gentamicin and amikacin, classical broad-spectrum antibiotics. This result is particularly relevant when considering the in vitro ability to inhibit the two selected *Staphylococcus* species—MSSA and MRSA. Among the conditions involved in the multifactorial etiopathogenesis of SD, the involvement of bacterial colonization is suggested by recent studies [4,20]. *Staphylococcus aureus* is not only indicated as most common bacterial member of the skin flora in patients with SD, but also as a microorganism able to trigger extensive and complex inflammatory response and elevated levels of antimicrobial resistance [20].

Previous studies pointed out antibacterial potential for distinct products derived from *K. lappacea* [21], *H. virginiana* [19], and *S. alba* [22]. Specifically, the *H. virginiana* extract proved to have an antiinflammatory and antibacterial effect on *Cutibacterium acnes*-induced inflammation in acne, whereas *K. lappacea* and *S. alba* showed important antibacterial activities toward a large number of bacterial strains, such as *Streptococcus pyogenes, Staphylococcus aureus, S. epidermidis,* and *Escherichia coli*. To the best of our knowledge, this is the first report indicating the potential therapeutical properties manifested by these three hydroalcoholic extracts’ combination. Taking all these into consideration and the fact that the chemical composition of these species is correlated with the antibacterial effect of each species, their combination appears to provide an interesting path to follow.

### 2.3. Cytotoxicity and Apoptosis Assay

**Figure 1:**
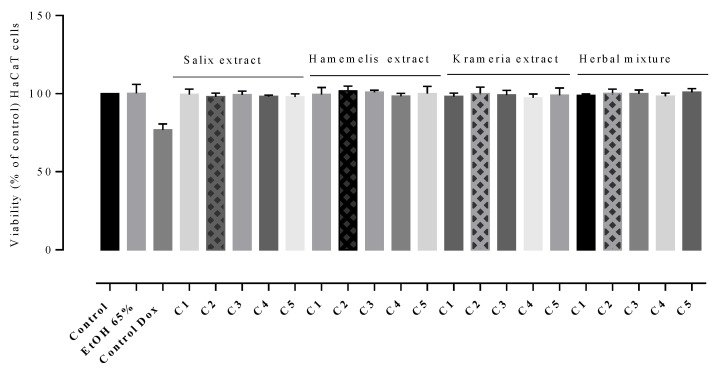
HaCaT cell line viability induced by the individual extracts and HM, evaluated at five concentrations (C1–C5) were established based on TPC values (mg GAE). The tested concentrations ranged between 0.0418 and 0.209 for *S. alba* extract, 0.0498 and 0.249 for *H. virginiana* extract, 0.0354 and 0.1761 for *K. lappacea* extract, and 0.0420 and 0.21 for HM. Negative control, untreated cells, Positive control, Doxorubicin (5 µM). Internal control, cells treated with 65% *v*/*v* ethanol solution (EtOH). Values represent the mean ± SD of three independent evaluations.

None of the tested concentrations of the hydroalcoholic extracts and HM expressed cytotoxic effects on human keratinocytes, the obtained viability percentages being close to those of the untreated cells (negative control). The range of HaCaT cell line viability was within 97.88% ± 2.04 and 99.45% ± 3.45 for *S. alba* (C5 = 0.209 mg GAE and C1 = 0.0418 mg GAE, respectively), 98.39% ± 1.86 and 101.85% ± 2.89 for *H. virginiana* (C4 = 0.1992 mg GAE and C2 = 0.0996 mg GAE, respectively), 97.37% ± 3.26 and 100.05% ± 4.08% for *K. lappacea* (C4 = 0.1416 mg GAE and C2 = 0.0708 mg GAE, respectively). The cell viability treated with HM ranged from 100.84% ± 0.006 (C1 = 0.0420 mg GAE) to 98.99% ± 0.006 (C5 = 0.21 mg GAE) (Figure 1). In fact, no significant differences were noticed between the viability percentages induced by the tested concentrations (C1–C5) compared to the negative control (*p* > 0.05).

Previous studies evaluated the individual cytotoxic effect effect of the three species used separately. It is only *H. virginiana* that has been tested for its antiinflammatory and anti-acne effects on human keratinocytes [19], showing a significant potential. For *S. alba* and *K. lappacea,* antiproliferative effects on human promyeloid leukemia cells [23] and on breast cancer cells were revealed [24].

**Figure 2:**
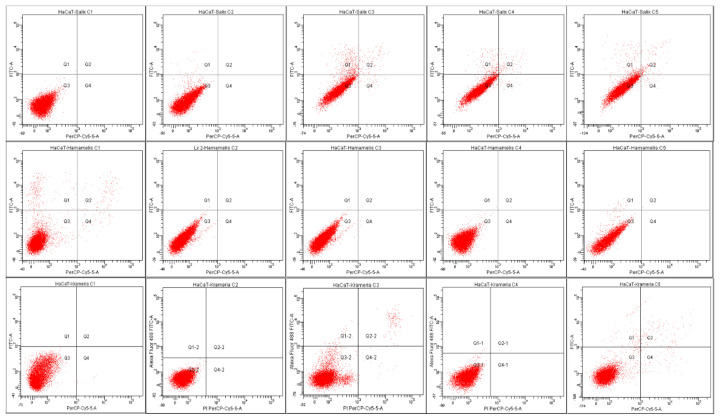
Apoptosis evaluation in HaCaT cells after treatment with hydroalcoholic extracts (*H. virginiana*, *S. alba*, *K. lappacea*). After 24 h of treatment, cells were stained with annexin V-FITC (50 µg/mL) and propidium iodide (100 µg/mL) and were evaluated with a BD FACS Canto II flow cytometer (Becton Dickinson, Franklin Lakes, New Jersey, USA), by using BD FACS Diva 6.1.2 software. The results are presented in scatter plots of annexin V-fluorescein isothiocyanate versus vital dye propidium iodide labelling Q3, viable cells; (annexin V-FITC (−), PI (−)); Q1, early apoptotic cells (annexin V-FITC (+), PI (−)), Q2, late apoptotic (annexin V-FITC (+), PI (+)); and Q4—necrotic cells (annexin V-FITC (−), PI (+)).

**Figure 3:**
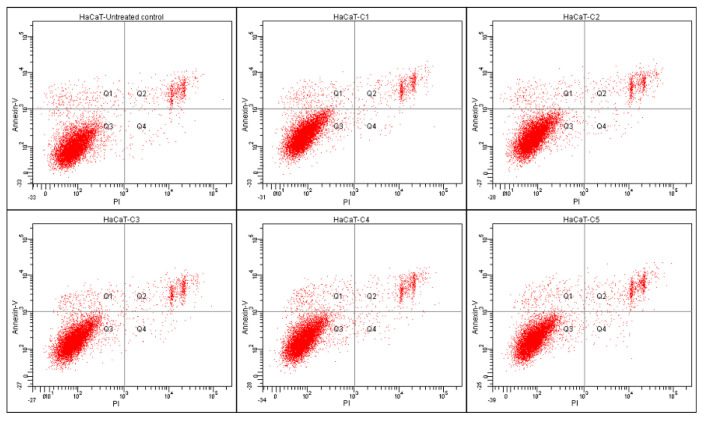
Apoptosis evaluation in HaCaT cells after treatment with the HM. After 24 h of treatment, cells were stained with annexin V-FITC (50 µg/mL) and propidium iodide (100 µg/mL) and were evaluated with a BD FACS Canto II flow cytometer (Becton Dickinson, USA), by using BD FACS Diva 6.1.2 software. The results are presented in scatter plots of annexin V-fluorescein isothiocyanate versus vital dye propidium iodide labelling Q3, viable cells; (annexin V-FITC (−), PI (−)); Q1, early apoptotic cells (annexin V-FITC (+), PI (−)); Q2, late apoptotic (annexin V-FITC (+), PI (+)); and Q4, necrotic cells (annexin V-FITC (−), PI (+)).

The viability results (MTT method) were further confirmed by flow cytometry followed by the Annexin V-FITC and PI assay (Figure 2 and Figure 3).

Apoptosis was measured by using flow cytometry. Figure 2 represents scatter plots of annexin V-fluorescein isothiocyanate (FITC-A) (y—axis) versus PI (PE-A) labelling (x—axes). Lower left quadrants indicate viable cells (absence of both markers); upper left quadrants (FITC-A positive, PE-A negative) indicate the apoptotic cells (early-stage apoptosis). The necrotic cells are represented by the right side of the panel (PE-A staining alone or together with FITC-A). None of the individual extracts in selected concentrations displayed inhibitory effect on HaCaT cell proliferation and the results were comparable to those obtained by using the MTT assay. A similar pattern was recorded for the HaCaT cells treated with HM in selected concentrations, with no effect on the viability and the cell condition being similar to the negative control (untreated cells). Whereas the average of the untreated cells’ viability was 90.8%, for the cells treated with the highest concentration of HM (C5 = 0.2l mg GAE), 89.5% maintained the viability after treatment, 2.6% of the cells were in early apoptosis, 7.3% of the cells were in late apoptosis, and 0.5% were represented by necrotic cells. It is the first report that assesses the apoptotic effect of the herbal combination and the first to report it on the selected species.

### 2.4. QbD Approach

The quality attributes (QAs), the critical material attributes (CMAs) and critical process parameters (CPPs) should be considered when defining a QTPP for a topical semisolid product. Thus, as presented in the Ishikawa diagram (Figure S3), qualitative and quantitative formulas of ingredients utilized as well as the manufacturing process factors are considered influencing the final emulsion properties [25]. Considering the risk score obtained for each CPP by using failure mode and effects analysis (FMEA) risk assessment (Table S3), the formulation factors chosen for the experimental design were the ratio of emulsifier, co-emulsifier, thickening agent, and oily phase.

#### 2.4.1. Experimental Design Matrix

The present study aimed to investigate the impact of the quantitative variation of the formulation factors, on the physical attributes of the oil-in-water creams. For this purpose, we have studied the influence of the emulsifier, co-emulsifier, thickening agent, and oily phase ratio on the emulsion characteristics: firmness, consistency, adhesiveness, stringiness, spreadability, and viscosity. The texture analysis has been chosen as an investigative tool for its recognized importance in characterization of high-quality cosmetic formulations, that meet consumers‘ acceptance.

To better understand the influence of the variables on the properties of the formulations, the DoE approach was used. DoE is a QbD tool, a mathematical methodology used for planning and conducting experiments, as well as for analysing and interpreting data obtained from experiments [26]. Lately, DoE was successfully implemented in the research and development of a cosmetics formulation based on natural ingredients by assessing the individual and interactive effects of raw materials on different responses related to the texture profile [27,28].

A D-optimal experimental design with four factors and two levels was developed. The selected factors were the ratio of emulsifier (X1), co-emulsifier (X2), and thickening agent (X3), and oily phase (X4). According to the experimental design matrix, 19 formulations were prepared, and the output responses, firmness (Y1), consistency (Y2), adhesiveness (Y3), stringiness (Y4), spreadability (Y5), and viscosity (Y6) were analyzed. Table 4 presents the matrix of the results obtained for the analysis of the 19 formulations from the experimental plan, expressed as mean ± standard deviation. Each analysis was performed in triplicate.

#### 2.4.2. Data Fitting and the Validation of the Experimental Plan

**Figure 4:**
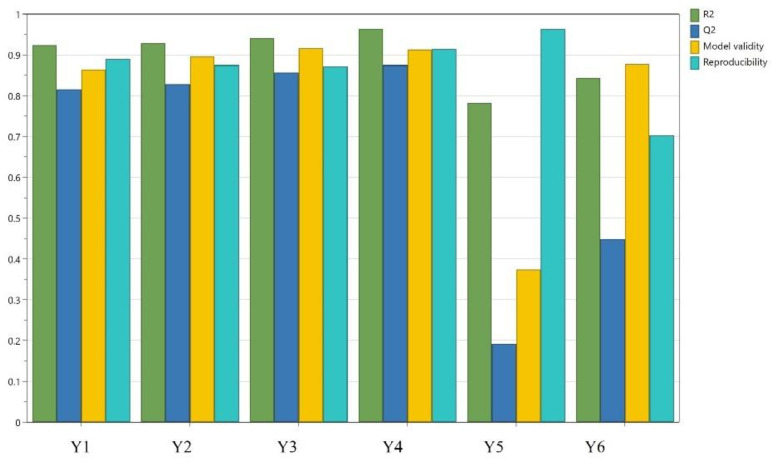
Summary of fit Y1, firmness; Y2, consistency; Y3, adhesiveness; Y4, stringiness; Y5, spreadability; Y6, viscosity.

According to Figure 4, the results obtained show an appropriate fitting of the experimental data with the chosen model. R^2^, the percent of the variation of the response explained by the model [27], indicates a good fit, with values above 0.7, which demonstrates a high significance of the model. The values of Q^2^, defined as the percent of the variation of the response predicted by the model [27], ranged between 0.23 and 0.98. Consequently, the high values of these two parameters indicate a good model. Furthermore, the difference between the two values should be smaller than 0.2–0.3, as smaller differences indicate an appropriately selected model. In this research, for all parameters except the Y5 (spreadability), the difference between R^2^ and Q^2^ showed an appropriately selected model. Together, both parameters R^2^ and Q^2^ are considered to be the most reliable statistical parameters that describe the validity of a model [13].

**Figure 5:**
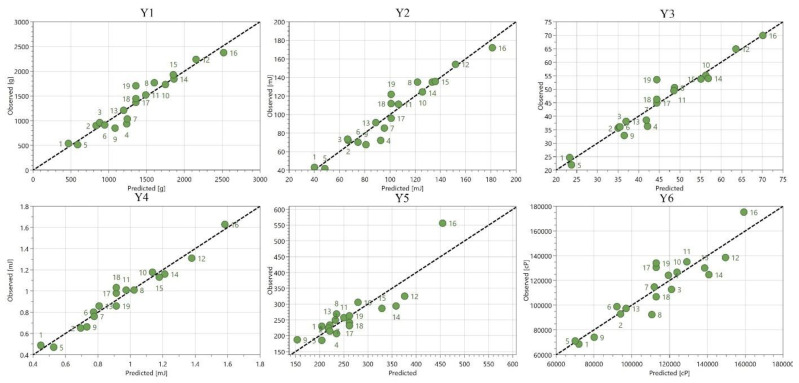
Residual curves of the observed responses as a function of the estimated responses (Y1, firmness; Y2, consistency; Y3, adhesiveness; Y4, stringiness; Y5, spreadability; Y6, viscosity).

The model validity parameter indicates whether the appropriate model type has been chosen. In this case, the validity was good for all answers [14]. The reproducibility, which reflects the variation of the response under the identical conditions, compared to the total variation of the response [27], reached high values, between 0.72 and 0.91. Table 5 presents the statistical parameters for the ANOVA test and the quality of fit. As observed in Table 5, the *p*-value was below 0.05, which indicates a statistically significant model. Figure 5 shows the residual curves of the observed responses as a function of the estimated responses. It is observed that the values follow close to the normal probability line. Therefore, the validity of the experimental plan was verified, and the results obtained after fitting the data proved to be good for all the studied answers.

#### 2.4.3. The Influence of the Formulation Factors on the Physical Characteristics of the Creams

**Figure 6:**
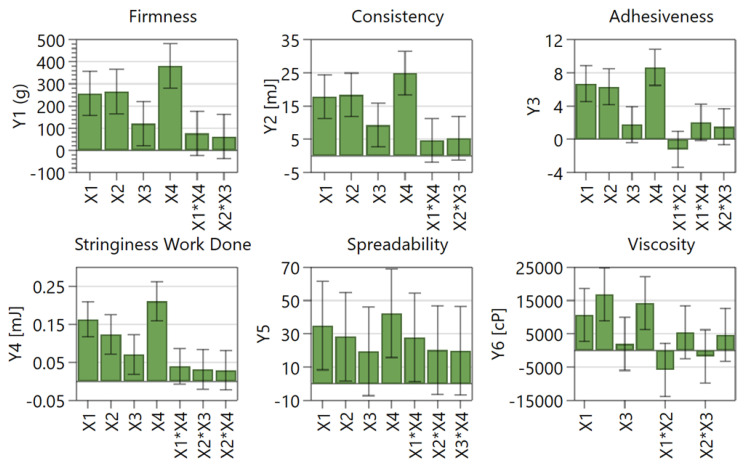
Coefficient plots (X1, emulsifier ratio, X2, co-emulsifier ratio, X3, thickening agent ratio, and X4, oily phase ratio; Y1, firmness; Y2, consistency; Y3, adhesiveness; Y4, stringiness; Y5, spreadability; Y6, viscosity).

Figure 6 represents the scaled and centered histograms which show the influence of the formulation factors represented by the input variables on the responses (output variables). These influences are also illustrated in Figure S7 as response surface plots. Only the significant influences are discussed further.

The firmness (Y1) of the creams varied between 521 g (N5) and 2370 g (N16). The firmness was positively influenced by the increase of the percentage of emulsifier, co-emulsifier, thickening agent, and oily phase. The greatest impact in increasing the firmness was obtained by increasing the percentage of oily phase, and the lowest influence in increasing this parameter was determined by increasing the percentage of the thickening agent.

The consistency (Y2) of the creams varied between 41.71 mJ (N5) and 171.80 mJ (N16). The input variables’ impact on consistency was very similar to the influence on the previous output parameter, the firmness. It is well known that the oily phase, containing in this case fatty compounds like cetylstearyl alcohol can increase the consistency of the emulsion. It also improves the texture of creams, increasing their viscosity depending on the amount added, and promotes skin moisturization [29,30]. Fatty alcohols, like cetylstearyl alcohol, can be used as thickening agent to increase the stability of the emulsions [31].

On the other side, the firmness and consistency of the hydrophilic phase were significantly influenced by Sepigel 305™ (INCI: polyacrylamide, C13-14 isoparaffin, laureth-7). This compound was incorporated into creams for its instantly gel-forming property in combination with water, but also for the ability to thicken, stabilize and texture the topical products [32].

The adhesiveness (Y3) varied between 21.97 (N5) and 69.95 (N16). The adhesiveness defines the sensory properties of cream and determines consumer acceptance [14]. In this case, the adhesiveness of the creams was increased by the addition of a high percentage of oily phase and emulsifiers.

The stringiness (Y4) varied between 0.47 mJ (N5) and 1.63 mJ (N16). The stringiness was positively influenced by the increase of the percentage of emulsifier, co-emulsifier, thickening agent, and oily phase, similarly to previous parameters.

The spreadability (Y5) was measured as the hardness of the sample recorded by using the TA-SF accessory. Spreadability improves as the hardness of the sample reduces. The spreadability values ranged between 184.3 (N5) and 556.8 (N16). This parameter is an attribute related to the performance and the perception of the formulation during its application to the skin, being defined as the ability of the formulation to cover the skin [33,34,35]. It depends, as can also be seen in this study, on the composition of the formulation [33,34]. More precisely, spreadability depends on the molecular weight, viscosity, and chemical structure of the ingredients [34]. Increasing both the emulsifier and the oily phase ratio, and their combined effect (X1*X4), led to an increase in the hardness value and indicates a less spreadable sample. The influence of emulsifiers and oily phase can be explained by their high viscosity and consistency.

The viscosity values ranged between 68,746 cP (N5) and 175,163 cP (N16). Similar to previous parameters, viscosity was positively influenced by the increasing ratio of emulsifier, co-emulsifier, and oily phase. The viscosity of the cream is important to ensure ease of application on the skin.

Therefore, the values of the studied parameters (firmness, consistency, adhesiveness, stringiness, spreadability, and viscosity) were increased by using a higher amount of emulsifier, co-emulsifier, and oily phase used in the formulation of the creams. Thus, these three formulation factors had a synergistic effect. The formulation N5 presented the lowest values of all analyzed parameters, while N16 had the greatest values. These are correlated with the lowest percentage of emulsifier, co-emulsifier, and oily phase used for the formulation of N5 and with the highest percentages of these components in N16. Interestingly, the percentage of the thickening agent was the same in both formulations, suggesting a greater influence of the emulsifiers and oily phase ratio than the thickening agent’s with regard to the texture and rheological properties. As was also reported in previous studies, the oily content of the formulations appears to have a high influence on their physical properties [36].

#### 2.4.4. Optimization—The Optimal Formulation

Both consumer acceptance and effectiveness of topical products involves optimized firmness, proper adhesiveness, and good spreadability. To obtain an optimal formula for stability and sensory characteristics, an optimization process was performed [14].

Based on the experimental results, a set of constraints were applied to the developed models of the most important responses defining the texture (firmness, consistency, adhesiveness, and viscosity), the composition of the optimal formulation being generated, as shown in Table 6. The optimal formulation was prepared experimentally and the practically obtained results are also presented in Table 6 together with the theoretical values provided by the optimization program.

According to these results, the experimental data showed a good correlation between the theoretical values predicted by the optimization program and the experimental values, thus confirming the validity of the proposed model.

### 2.5. General Overview of the Optimal Formulation

Apart from the HM, the other ingredients of the cream were carefully chosen to restore the impaired skin barrier but also to confer good sensory properties of the dermocosmetic product, a favourable texture, and noncomedogenic characteristics.

Among many effects, allantoin is recognized for its skin-protectant, moisturizing and keratolytic properties. Allantoin has also been found to have wound-healing properties by helping to proliferate epithelial cells and debriding the necrotic tissue [37].

Urea, a component of the skin’s natural moisturizing factor (NMF), plays an important role in preserving skin hydration and integrity [38]. At low concentrations (≤10%), urea can change the structure of proteins, especially keratin, increasing the ability to bind water and hydration, while at high concentrations (>10%), it has keratolytic action, practically dissolving keratin. In general, creams containing low concentrations of urea can restore the skin barrier and topical use of urea increases lipid biosynthesis [39].

Additionally, glycerol has powerful moisturizing properties, helps to maintain water in the upper layers of the skin, and maintains skin’s softness [29]. Glycerine is involved in the desquamation process, which is a critical phase of the skin renewal cycle, through its ability to enhance the digestion of desmosomes [40].

In addition to hydrophilic active ingredients, lipid emollients were added to the cream to improve skin moisturization and restore the lipid barrier of the stratum corneum. Avocado oil (*Persea gratissima* oil) extracted from the pulp of *P. gratissima* fruit is rich in polyunsaturated oily acids (PUFA), linoleic and linolenic acids, and monounsaturated oily acids represented by oleic acid. It also contains β-sitosterol, β-carotene, lecithin, minerals, and vitamins [41]. It is a yellow-green liquid with medium viscosity and a characteristic odour. Avocado oil is regenerating, restructuring, emollient, acts against skin aging, softens and maintain the elasticity of the skin, and attenuates inflammatory reactions [29].

Almond oil (*Prunus amygdalus dulcis* oil) is obtained by the cold-pressing technique from the seeds of the *Prunus amygdalus* var. *dulcis* (Borkh. ex DC.) Koehne tree. Almond oil is rich in oleic acid, linoleic acid (ω-6), stearic acid, palmitic acid, phytosterols, and triterpene alcohols. The application of almond oil on the skin has a moisturizing and emollient long-lasting effect, which helps to maintain the integrity of the skin barrier [29,42].

Argan oil *(Argania spinosa* kernel oil) is obtained by the cold-pressing technique from the kernel of the *A. spinosa* fruit. Argan oil contains mainly triacylglycerols, monoacylglycerols, diacylglycerols, and free oily acids, the minor components being polyphenols, sterols, tocopherols, triterpene alcohols, and squalene [43]. Argan oil has been shown to have an antiaging effect due to its properties that improve skin elasticity [44].

Caprylic/capric triglycerides are components of the oily phase of creams, produced by esterifying glycerol with mixtures of caprylic and capric acids from the oily acids of coconut or palm oil. The resulting triglycerides improve the texture of the cosmetics, decrease the viscosity, and due to their antioxidant properties, prolong the stability of the compounds [45].

Coco-caprylate is a derivative of plant origin which confers a pleasant, silky, and nongreasy feeling when applied topically. It has emollient properties and maintains the integrity of the epidermal barrier [29].

In summary, it can be concluded that the developed oil-in-water cream fulfils adequate characteristics in terms of functional benefits, argued by the careful choice of ingredients, but also favourable texture and sensory attributes. Future studies will be focused on the evaluation of the in vivo skin performances of the developed product.

## 3. Materials and Methods

### 3.1. The Individual Extracts and HM Preparation

The HM was prepared as a combination of equal parts of three individual extracts obtained as follows.

- *H. virginiana* extract was obtained from the dried crushed leaves of *H. virginiana*, native to Oregon, USA (provided by Galke GmbH, Germany, article no. 60002). The finely chopped leaves were extracted with 50% *v*/*v* ethanol in water, in a 1:6 extraction ratio. The cold extraction was performed by maceration under periodical stirring, for 10 days and according to method 1.1.8 of the European Pharmacopoeia, 10th edition [46]. Finally, the extractive solution was decanted and then filtered after five days.
- *S. alba* extract was obtained from the fresh bark of *S. alba* collected from Rădaia, Cluj County, Romania (latitude 46°48′05.54″ N, 23°27′51.62″ E, voucher no. 65319). The extraction was performed with 90% *v*/*v* ethanol in water. Considering the humidity of the plant material (58.36%), the extraction ratio was 1:1.2 for the fresh herbal material, respectively 1:2 for the dried herbal material. The cold extraction was performed by maceration under periodical stirring, for 10 days, according to method 1.1.5 of the European Pharmacopoeia, 10th edition [46]. Finally, the extractive solution was decanted, and the solid material was pressed to collect the residual extract, followed by the mixing of the two solutions. After five days the tincture was filtered.
- *Ratanhia* extract was obtained from the dried crushed root of *K. lappacea*. The plant native Peru was provided by Galke GmbH (Bad Grund, Germany, article no. 101802). The crushed root was extracted with 70% *v*/*v* ethanol in water, in a 1:9 extraction ratio. The cold extraction was performed by maceration under periodic stirring for 10 days, according to a method described in the 10th edition of the European Pharmacopoeia [46]. The extract was filtered after five days.
- The vegetal material was verified and identified by Lecturer Irina Ielciu, PhD. For the *K. lappacea* and *H. virginiana* species, commercial products were used, whereas for *S. alba*, voucher specimens of the harvested vegetal material are preserved at the Department of Quality Controle at the PlantExtrakt Ltd. (Rădaia, Cluj-Napoca, northwestern Romania).
- Alcoholic concentration of the HM was measured by using an official method of the 10th edition of the European Pharmacopoeia and was established at 65% and the extraction yield was established at 74.5%.

Afterward, the total polyphenolic content of the extracts was assessed according to the method described in European Pharmacopoeia 10th edition by using the Folin-Ciocâlteu reagent [46,47,48]. The absorbances of the samples were measured spectrophotometrically at 760 nm. The total polyphenolic content was expressed as mg gallic acid equivalent (mg GAE/mL extract).

The phytochemical composition and the biological activities were assessed on the individual extracts and on the HM. In the last step, the HM was concentrated by using a rotary evaporation (Hei-VAP Advantage Rotary evaporator HL/G1; Heidolph, Schwabach, Germany) at 60 °C, 40 rpm, and 250 mbar until 25% of the initial amount. The HM was subsequently incorporated into the oil-in-water cream.

### 3.2. LC/MS Method

The LC/MS apparatus consisted in a Shimadzu Nexera I LC/MS-8045 (Kyoto, Japan) UHPLC system, equipped with a quaternary pump and autosampler, respectively, an ESI probe, and quadrupole rod mass spectrometer. This method was validated according to International Conference on Harmonization (ICH) guidelines.

Separation was achieved on a Luna C18 reversed phase column (150 mm × 4.6 mm × 3 mm, 100 Å), from Phenomenex (Torrance, CA, USA). The column was maintained at 40 °C degrees during the analysis.

The mobile phase (Table S2) consisted of a gradient obtained from methanol (Merck, Darmstadt, Germany) and ultrapurified water prepared by Simplicity Ultra Pure Water Purification System (Merck Millipore, Billerica, MA, USA). As an organic modifier, formic acid was used (Merck, Darmstadt, Germany). The methanol and the formic acid were of LC/MS grade. The used flow rate was 0.5 mL/min. The total analysis time was 35 min.

The detection was carried out on a quadrupole rod mass spectrometer operating with electrospray ionization (ESI), in negative and positive multiple reaction monitoring (MRM) ion mode. The interface temperature was maintained at 300 °C degrees. For vaporization and as a drying gas, nitrogen was used at 35 psi, respectively, at 10 mL/min. The capillary potential was set at +3000 V.

Identification was achieved by comparison of the retention times, MS spectra and the main transitions between compounds from the tested extracts and references (Table 7 and Table 8). Identification and quantification were performed based on the main transition from the MS spectra of each compound. Calibration curves (R^2^ = 0.9964–0.9999) were determined for the quantification of the compounds. For the tested extracts, 10 μL were injected after dilution 1:10 with methanol and for the references, 1 μL were injected [49,50].

### 3.3. Antimicrobial Activity Evaluation

The in vitro antimicrobial potential was initially screened by using the agar well diffusion assay, a modified European Committee on Antimicrobial Susceptibility Testing (EUCAST) [51] disk-diffusion method. Seven bacterial reference strains obtained from Oxoid Ltd. (Hampshire, UK) were considered, namely *Staphylococcus aureus* ATCC 25923 (methicillin-susceptible *S. aureus*, MSSA), *Staphylococcus aureus* ATCC 700699 (methicillin-resistant *S. aureus*, MRSA), *Bacillus cereus* ATCC 14579, *Enterococcus faecalis* ATCC 29219, *Escherichia coli* ATCC 25922, *Salmonella enterica* serovar Typhimurium ATCC 14028, and *Pseudomonas aeruginosa* ATCC 27853. The culture media, Mueller Hinton (MH) broth and agar, were purchased from Merck (Darmstadt, Germany, catalogue number 70192 and 70191-500G). For each organism, the bacterial inoculum prepared by suspending 24 h pure culture in Mueller Hinton (MH) broth to obtain 1.5 × 10^8^ colony-forming unit (CFU)/mL, according to the McFarland scale, was “flood-inoculated” on MH agar plates; six-millimeter diameter wells (three for each sample) aseptically made into the MH agar were filled with 60 μL of tested product and 65% ethanol in water *v*/*v*, respectively (as the negative control). Gentamicin (10 µg), amoxicillin-clavulanic acid (20–10 µg), amikacin (30 µg) disks (Oxoid Ltd., Hampshire, UK, catalogue number CT0794B, CT0223B and CT0107B, respectively) were included as standard antibiotics. Following 24 h of incubation at 37 °C, the growth inhibition zone diameters (in mm) were measured. Minimum inhibitory (MIC) and bactericidal (MBC) concentrations were also established by using a broth microdilution method. As MIC positive and negative controls, gentamicin stock solution 50 mg/mL in sterile deionized water (Sigma-Aldrich, St. Louis, MO, USA) and MH broth, respectively, were tested. Briefly, twofold serial dilutions of the tested product were performed in 100 µL MH broth; 5.0 µL of a 24 h 1.5 × 10^8^ CFU/mL bacterial inoculum were added to each well and incubated for 24 h at 37 °C. MIC values represented the lowest concentrations able to inhibit the visible bacterial growth of (no turbidity in the well) when compared to the negative control (MH broth); 10.0 µL from each well were further cultured on MH agar plates for 24 h at 37 °C to allow the reading of MBCs values as the lowest concentrations associated with no visible bacterial growth on the MH agar plates. The testing was performed in duplicate. Furthermore, based on the ratio MBC/MIC, the MIC index was also calculated to evaluate whether the extract exhibits bactericidal (MBC/MIC ≤ 4) or bacteriostatic (MBC/MIC > 4) effect against the tested bacterial strains [50,52].

### 3.4. Cytotoxicity and Apoptosis Assay

The potential cytotoxicity of the hydroalcoholic extracts and of the HM was assessed on human keratinocyte (HaCaT, ATCC) cells, a standardized cell line obtained from the “Ion Chiricuţă” Institute of Oncology (Cluj-Napoca, Romania). The cells were maintained in Dulbecco’s Modified Eagle’s Medium (DMEM) (Sigma-Aldrich, St. Louis, MO, USA) with 10% fetal bovine serum (Gibco Life Technologies, Paisley, UK) and 1% antibiotic/antimycotic (Gibco Life Technologies, Paisley, UK) at 37 °C with 5% CO_2_. The cytotoxic effects were evaluated with MTT assay (3-(4,5-dimethyl-2-thiazolyl)-2,5-diphenyl-2H-tetrazolium bromide). The cultures were treated with 0.25% trypsin-EDTA, to obtain cell suspensions, and following centrifugation (1500 rpm for 5 min), 1 × 10^4^ cells/well were seeded on 96-well plates in 200 µL complete culture medium. The individual extracts and HM were added in five different volumes (5, 10, 15, 20, 25 µL) with the resulting concentrations established based on the determined total phenolic content (TPC) and expressed as mg GAE as follows: *S. alba* extract (C1 = 0.0418, C2 = 0.0836, C3 = 0.1254, C4 = 0.1672, C5 = 0.209 mg GAE), *H. virginiana* extract (C1 = 0.0498, C2 = 0.0996, C3 = 0.1494, C4 = 0.1992, C5 = 0.249 mg GAE), *K. lappacea* extract (C1 = 0.0354, C2 = 0.0708, C3 = 0.1062, C4 = 0.1416, C5 = 0.1761 mg GAE), and HM (C1 = 0.0420, C2 = 0.084, C3 = 0.126, C4 = 0.168, C5 = 0.21 mg GAE).

The negative control was represented by untreated cells maintained in a normal propagation medium. The internal control was represented by cells treated with 65% ethanol. The positive control was represented by doxorubicin (Merck, Darmstadt, Germany)-treated cells. Each experimental condition was performed in triplicate. After 24 h, the medium was removed and 100 µL of 1 mg/mL MTT solution (Sigma-Aldrich, St. Louis, MO, USA) was added. After 3 h of incubation at 37 °C in dark, the MTT solution was removed from each well and 150 µL of DMSO (dimethyl sulfoxide) solution (Fluka, Buchs, Switzerland) was added. Spectrophotometric readings at 450 nm were performed with a BioTek Synergy 2 microplate reader (Winooski, VT, USA). Data are shown as optical density or average proliferation percentage rate compared with the control of untreated cells [53].

The Annexin V-FITC Apoptosis Detection Kit (Sigma-Aldrich, St. Louis, USA) was used to determine the apoptosis. The HaCaT cells were stained with annexin V and propidium iodide solution (PI) according to the kit’s instructions, and the fluorescent intensity was read with a BD FACS Canto II flux cytometer (Becton Dickinson, USA), with a two-laser configuration: 20 mW argon solid state, at 488 nm, and 17 mW neon-helium (NeHe), at 633 nm. The flow cytometer was programmed to capture information from the corresponding photodetector for annexin V (FL1-A) and PI (FL3-A), on a logarithmic scale. Initially, an unstained control sample was analyzed for FSC-A (forward scatter) and SSC-A (side scatter) signals to identify the cell population of interest and remove debris. The fluid pressure was set to a minimum, so the acquisition speed was appropriate. Subsequently, the stained samples were read. Fluorescence detection was achieved with a 488-nm laser and the 525/50 filter for annexin V and 695/40 filter for PI. 10,000 events (cells) were examined for each sample. Results analysis was performed with the BD FACS Diva 6.1.2 software. Thus, fluorescence intensity was presented in dot plots, each being divided into four quadrants. Cells that did not stain with either of the fluorescent dyes appeared in quadrant 3 (Q3-viable cells). Cells that were only stained with annexin appeared in quadrant 1 (Q1-apoptotic cells), and cells that stained with both annexin and PI appeared in quadrant 2 (Q2-late apoptotic cells). Cells in quadrant 4 (Q4) are only stained with PI and therefore are necrotic cells. A total of 10.000 cells were analyzed and included in each dot plot [50].

### 3.5. Obtention of the Oil-in-Water Cream

#### 3.5.1. Materials for Cream Preparation

The ingredients used for the formulation were allantoin, urea, glycerine (Elemental, Oradea, Romania), Sepigel™305 (polyacrylamide, C13-14 isoparaffins, Laureth-7; Seppic, Paris, France), Euxyl PE 9010 (phenoxyethanol, ethylhexylglycerin; Schulke& Mayr, Norderstedt, Germany), cetylstearyl alcohol (Vitamar, Bucharest, Romania), caprylic/capric triglycerides (Croda, Snaith, UK), coco–caprylate, avocado oil (*Persea gratissima* oil, almond oil (*Prunus amygdalus dulcis* oil), argan oil (*Argania spinosa* kernel oil), Emulgade^®^ Sucro (sucrose polystearate, hydrogenated polyisobutene) and cetearyl glucoside (Elemental, Oradea, Romania).

#### 3.5.2. Oil-in-Water Cream Preparation

The aqueous phase was prepared by adding cetearyl glucoside, Sepigel™305, urea, allantoin, and glycerin to the necessary amount of water and maintained on the water bath at 65 °C (±2 °C). Separately, cetylstearyl alcohol and Emulgade^®^ Sucro were heated to melt, then sequentially added the liquid components, caprylic/capric triglycerides, coco-caprylate, avocado oil, almond oil, and argan oil under continuous stirring, at 65 °C (±2 °C). The oily phase was gradually added to the aqueous phase while mixing with the Ultra-turaxx T50 device (Heidolph, Germany) at 1000 rpm for 15 min and gradually cooled. To avoid the exacerbation of skin irritation induced by ethanol, the extract was concentrated to 25% of the initial amount in a rotary evaporator and then incorporated into the oil-in-water cream in a 10% ratio. The HM was added under continuous stirring at low speed for another 10 min until a homogenous product was obtained.

### 3.6. Characterization of the Oil-in-Water Cream

In recent years, increased attention has been focused on the exhaustive evaluation of cosmetic products to optimize the performance of the formulations and also to ensure an optimal sensory profile. In this sense, specific tests have been developed for the complete assessment of those products. Thus, specific sensorial characteristics can be predicted by interpretation of the physical-mechanical parameters recorded through rheological and texture analysis measurements [14,54,55].

The CT3 (Brookfield Engineering Laboratories, Middleboro, MA, USA) Texture Analyzer was used to determine the texture of the creams. The following parameters were determined by using the TA-DEC probe: firmness, consistency, adhesiveness, and by using the TA-SF configuration: spreadability, and stringiness work done. The TA-BT-KIT fixture system was used in both configurations. For each of the 19 formulations, three measurements were performed, and the mean value was reported. The results were recorded by using Texture ProCT Software 1.9 (Brookfield Engineering Laboratories, Middleboro, MA, USA).

The viscosity was determined by using the Brookfield DV III Ultra Rheometer (Brookfield Engineering Laboratories, Middleboro, MA, USA) equipped with an LV-4 spindle. Experimental data were acquired by Rheocalc^®^ software at 24 °C within a rotational speed of 1–5 rpm. Apparent viscosity recorded at a testing speed of 2 rpm was integrated and analyzed within DoE.

### 3.7. QbD Approach

#### 3.7.1. Definition of QTPP, CQAs

According to the QbD approach, the first step in the development of the cream was the establishment of a quality target product profile (QTPP) and the quality attributes (QAs) that are required to ensure the desired performance of the product [25,56,57]. The QTPP for oral dosage forms is well defined, while QTPP for topical semisolid products has not been established yet; thus, these attributes will vary relative to the different categories of topical products [25]. Cosmetics regulation requirements are less exigent than those in pharmaceutical industry [58] and a large number of products with highly diverse features can be marketed. However, the cosmetics marketing companies have the legal obligation to grant the safety of the products. The general aim of the development of a cosmetic formulation is to obtain a stable product with sensory features in agreement with the purpose of the formulation. These quality characteristics are better observed, understood, and acquired by using the QbD approach.

The QTPP was established to achieve the desired cream quality, represented by the following characteristics: optimized firmness, proper adhesiveness, good spreadability, and proper viscosity. All these characteristics were chosen to obtain a favourable sensory profile for increased patients’ acceptability, taking into consideration the longer period of use in seborrheic dermatitis. Table 9 contains the QTPP and QAs considered relevant for this study, which were established based on previously reported studies that use similar settings [14,59].

#### 3.7.2. Risk Analysis

The Ishikawa diagram and the FMEA approach were used to perform a risk assessment, which allowed the identification and analysis of risk variables for the preparation of the developed oil-in-water emulsion. FMEA is based on evaluation of three criteria: frequency of occurrence (O), severity of consequences (S), and difficulty of detection (D). Each of these criteria was attributed to each CPP (formulation factors and process parameters) and was ranked from 1 to 5 on a scale: the occurrence (O) was evaluated as 5 for frequent, 4 for probable, 3 for occasional, 2 for remote, and 1 for improbable; the severity (S), was ranked as 5 for catastrophic, 4 for critical, 3 for serious, 2 for minor, and 1 for negligible; detectability (D) was given a score of 5 for hard to detect, 4 for low chance of detection, 3 for moderately detectable, 2 for highly detectable, and 1 for easily detectable. The risk score has been revealed as risk priority number (RPN), by multiplying these three attributes [59].

#### 3.7.3. Experimental Design

To assess the effect of variables and determine which of those variables has the greatest impact on the final properties of the formulation, a design of experiements (DoE) approach was used. The Modde 12 software (Sartorius Stedim, Goettingen, Germany) was used to develop the DoE and to carry out the statistical analysis of the collected data. A D-optimal experimental design with four factors and two levels was conceived to identify the effects of key ingredients, on the physical characteristics of the developed creams. The four independent variables together with their main functions are presented in Table 10. Nineteen formulations were prepared, and the output responses (firmness (Y1), consistency (Y2), adhesiveness (Y3), stringiness (Y4), spreadability (Y5), and viscosity (Y6)) were analyzed according to the applied design (Table 11).

### 3.8. Statistical Analysis

The results are presented as mean ± standard deviation (SD). The data from the in vitro tests were statistically analyzed by using ANOVA GraphPad Prism software, version 6.0 (San Diego, CA, USA). One-way analysis of variance (ANOVA) was performed, followed by Tukey’s post hoc test, to establish the statistical significance between means of the antimicrobial assays and cytotoxicity evaluation. The Modde 12 software was used to perform data processing of the nineteen formulations from the experimental plan. The data was statistically analyzed by ANOVA. A *p*-value below 0.05 was considered statistically significant.

## 4. Conclusions

The phytochemical analyses performed on the individual extracts and on their mixture bring important arguments for their combination, with the polyphenolic profile enrichment, proving synergistic antimicrobial effects. The present study reports, therefore, for the first time the antibacterial effect of a *K. lappacea* roots, *H. virginiana* leaves, and *S. alba* bark herbal extracts combination on various bacterial strains. At the same time, the obtained HM proved no cytotoxic effect on human keratinocytes, bringing further arguments for the safety and efficacy of its topical administration.

Moreover, the study describes a QbD-based development of topical products containing an innovative combination of hydroalcoholic extracts together with restructuring and soothing agents.

To study the impact of the formulation factors on the characteristics of the cream, a D-optimal experimental plan with four factors and two levels was used. Nineteen different cream formulations were generated by the software by varying the ratio of emulsifier, co-emulsifier, thickening agent, and oily phase (input variables). The effects of the quantitative variation of these formulation factors on the output variables were studied. The output variables were represented by firmness, consistency, adhesiveness, stringiness, spreadability, and viscosity of the formulations.

After the analysis of the prepared formulations, it could be concluded that both the emulsifier and co-emulsifier ratio and the high ratio of lipophilic phase have a significant influence on the quality of the prepared creams. The increase in the percentage of emulsifier (sucrose polystearate and hydrogenated polyisobutene), the co-emulsifier (cetearyl glucoside), and the oily phase (cetylstearyl alcohol, caprylic/capric triglycerides, coco-caprylate, avocado oil, argan oil, and almond oil) led to an increase of values for all studied parameters.

Based on this study, an optimal formulation was obtained with appropriate properties in terms of firmness, consistency, adhesiveness, stringiness, tensile capacity, and viscosity. The optimal formulation was prepared and analyzed, and the results obtained were close to those generated by the experimental plan.

This approach allowed us to obtain a novel herb-based oil-in-water cream with appropriate composition and optimal sensory attributes, that may be proposed for managing seborrheic dermatitis symptoms.

## Figures and Tables

**Table 1:** LC/MS identification and quantification (mg/mL) of polyphenols in the composition of the individual extracts and HM.

Identified Compound	Retention Time (min)	*m*/*z* and Main Transition	*H. virginiana* Leaves Extract	*K. lappacea* Roots Extract	*S. alba* Bark Extract	HM
Caffeic acid	13.8	179.0 > 135.0	1.698 ± 0.0248	2.310 ± 0.0488	<LoQ	1.392 ± 0.0696
Carnosic accid	32.0	331.2 > 285	0.918 ± 0.0174	2232 ± 0.0708	0.909 ± 0.0119	1.237 ± 0.0630
Chlorogenic acid	11.9	353.0 > 191.0	20.615 ± 0.2478	17.145 ± 0.2864	0.189 ± 0.0047	12.604 ± 0.2898
Ferulic acid	18.4	193.0 > 134.0	<LoQ	<LoQ	0.339 ± 0.0041	0.117 ± 0.0069
Gallic acid	7.0	168.0 > 125.0	6.790 ± 0.0453	0.088 ± 0.0005	<LoQ	2.306 ± 0.1120
Ellagic acid		<LoQ	1.119 ± 0.0094	<LoQ	<LoQ	0.377 ± 0.0127
Salicylic acid	23.5	137.0 > 93.0	<LoQ	<LoQ	11.993 ± 0.0921	4.169 ± 0.1459
*trans*-p coumaric acid	17.4	163.0 > 119.0	2.480 ± 0.0083	<LoQ	<LoQ	0.796 ± 0.0461
Apigenin	28.1	269.0 > 117.0	0.010 ± 0.0001	0.040 ± 0.0001	0.760 ± 0.0035	0.289 ± 0.0167
Catechin	10.3	289.0 > 202.9	0.105 ± 0.0009	<LoQ	<LoQ	0.038 ± 0.0024
Chrysin	29.7	253.0 > 143.0	0.117 ± 0.0009	0.059 ± 0.0002	0.319 ± 0.0051	0.172 ± 0.0101
Hyperoside	20.4	463.1 > 300.0	31.600 ± 0.2481	0.300 ± 0.0042	0.175 ± 0.0029	10.506 ± 0.2206
Kaempferol	27.9	285.0 > 187.0	1.400 ± 0.0264	0.053 ± 0.0004	0.070 ± 0.0005	0.530 ± 0.0307
Luteolin	26.8	287.0 > 153.0	0.076 ± 0.0002	0.005 ± 0.0001	<LoQ	0.026 ± 0.0017
Luteolin-*7-O*-glucoside	19.9	447.0 > 284.9	0.225 ± 0.0034	0.055 ± 0.0001	0.057 ± 0.0001	0.105 ± 0.0061
Myricetin	13.6	317.0 > 137.0	1.639 ± 0.0291	6.366 ± 0.0579	7.656 ± 0.0943	5.164 ± 0.1549
Naringenin	26.3	271.0 > 119.0	0.240 ± 0.0015	0.420 ± 0.0012	143.820 ± 2.943	49.670 ± 0.5463
Pyrocatechol	11.7	109.0 > 90.6	<LoQ	0.090 ± 0.0001	3.968 ± 0.0721	1.470 ± 0.0735
Quercetin	25.4	300.9 > 151.0	10.112 ± 0.2246	0.040 ± 0.0001	0.020 ± 0.0001	3.242 ± 0.1199
Quercitrin	22.1	447.0 > 229.9	1.269 ± 0.0397	<LoQ	<LoQ	0.455 ± 0.0268
Rutoside	20.2	609.0 > 300.0	21.525 ± 0.4293	0.345 ± 0.0045	<LoQ	7.363 ± 0.2208
Vitexin	18.4	431.0 > 311.0	0.045 ± 0.0001	0.055 ± 0.0001	<LoQ	0.034 ± 0.0002

Note: Values represent the mean ± standard deviation of three independent measurements. <LoQ—identified, but not quantified (below limit of quantification).

**Table 2:** In vitro antibacterial activity of the individual extracts and of the HM (agar well-diffusion assay).

	Zone of Inhibition (mm)						
**Reference Strains**	***S. alba* Bark Extract**	***H. virginiana* Leaves Extract**	***K. lappacea* Roots Extract**	**HM**	**Amoxicillin- Clavulanic**	**Gentamicin**	**Amikacin**
MSSA	19.67 ± 0.52	19.17 ± 0.4	18.5 ± 0.84	21.33 ± 1.03 ^a^	29 ± 0.00 ^a,b^	20 ± 0.00	21 ± 0.00
MRSA	14.50 ± 0.55	16.83 ± 0.41	17 ± 0.63	18.25 ± 1.21 ^a^	28 ± 0.00 ^a,b^	17 ± 0.00	21 ± 0.00
*Bacillus cereus*	15.17 ± 0.41	18.17 ± 0.41	18.17 ± 0.41	21.00 ± 0.00 ^a^	17 ± 0.00	21 ± 0.00	18 ± 0.00
*Enterococcus* *faecalis*	18.33 ± 0.52	17.67 ± 0.52	16.33 ± 0.52	18.33 ± 0.52 ^a^	15 ± 0.00	0	0
*Salmonella* *enterica* serovar Enteriditis	9.67 ± 0.52	10.83 ± 0.41	8.67 ± 0.52 ^a^	14.50 ± 0.55 ^a^	18 ± 0.00 ^a,b^	18 ± 0.00 ^a,b^	18 ± 0.00 ^a,b^
*Escherichia coli*	10 ± 0.52	8.5 ± 0.52	10.75 ± 0.41	14.25 ± 0.50 ^a^	19 ± 0.00 ^a,b^	19 ± 0.00 ^a,b^	19 ± 0.00 ^a,b^
*Pseudomonas* *aeruginosa*	0	0	0	0	0	18 ± 0.00	21 ± 0.00

Note: MSSA, methicillin-susceptible *Staphylococcus aureus*; MRSA, methicillin-resistant *Staphylococcus aureus.* Values represent the mean ± standard deviations of two independent measurements. ^a,b^ Means with different subscript letters within a column are significantly different at *p* < 0.05; HM (8.41 mg GAE/mL). Antibiotic disks: Amoxicillin-clavulanic (20–10 µg), Gentamicin (10 µg), Amikacin (30 µg).

**Table 3:** In vitro antibacterial activity of the individual extracts and of the HM (broth microdilution method).

Samples	Parameters	Reference Strains					
		MSSA	MRSA	*Bacillus cereus*	*Enterococcus faecalis*	*Salmonella enterica* Serovar Enteriditis	*Escherichia* *coli*
*Salix alba*	MIC (mg GAE/µL)	0.1047	0.2094	0.1047	0.4188	0.4188	0.4188
	MBC (mg GAE/µL)	0.4188	0.4188	0.4188	0.4188	0.4188	0.4188
	MIC index MBC/MIC	3	2	3	1	1	1
*Hamamelis virginiana*	MIC (mg GAE/µL)	0.2494	0.2494	0.2494	0.4988	0.4988	0.4988
	MBC (mg GAE/µL)	0.4988	0.4988	0.4988	0.4988	0.4988	0.4988
	MIC index MBC/MIC	2	2	2	1	1	1
*Krameria lappacea*	MIC (mg GAE/µL)	0.1774	0.3548	0.1774	0.3548	0.3548	0.3548
	MBC (mg GAE/µL)	0.3548	0.3548	0.3548	0.3548	0.3548	0.3548
	MIC index MBC/MIC	2	1	2	1	1	1
HM	MIC (mg GAE/µL)	0.12	0.25	0.25	0.49	0.49	025
	MBC (mg GAE/µL)	0.25	0.25	0.25	0.49	0.49	0.49
	MIC index MBC/MIC	2	1	1	1	1	2
Gentamicin	MIC (mg/L)	3	4	3	-	2	2

**Table 4:** The results of selected output responses.

Experiment Name	Y1	Y2	Y3	Y4	Y5	Y6
N1	542.8 ± 26.50	43.03 ± 4.49	24.58 ± 3.36	0.49 ± 0.22	230.7 ± 69.60	68,476 ± 5738.78
N2	896.8 ± 38.40	71.97 ± 3.37	35.56 ± 0.64	0.65 ± 0.04	219.8 ± 20.00	92,750 ± 7677.22
N3	959.0 ± 48.90	73.30 ± 4.78	35.97 ± 1.70	0.67 ± 0.06	234.7 ± 3.70	112,676 ± 7029.85
N4	930.7 ± 76.30	72.04 ± 6.82	36.25 ± 1.38	0.70 ± 0.06	207.5 ± 0.04	124,373 ± 10,774.06
N5	521.0 ± 35.00	41.71 ± 3.94	21.97 ± 1.02	0.47 ± 0.03	184.3 ± 7.80	70,884 ± 5110.66
N6	914.8 ± 14.30	70.21 ± 1.29	36.01 ± 0.43	0.80 ± 0.04	224.0 ± 18.30	98,778 ± 2251.18
N7	1042.0 ± 14.90	85.33 ± 1.20	38.62 ± 0.38	0.76 ± 0.06	213.5 ± 11.40	114,676 ± 3868.28
N8	1765.0 ± 24.30	135.00 ± 7.60	50.53 ± 0.58	1.01 ± 0.06	268.0 ± 17.80	92,180 ± 6532.36
N9	850.5 ± 21.10	67.24 ± 6.54	32.86 ± 1.56	0.66 ± 0.12	186.5 ± 15.50	74,184 ± 4502.37
N10	1725.0 ± 45.50	124.40 ± 8.77	55.08 ± 0.90	1.18 ± 0.01	305.2 ± 4.30	126,573 ± 2399.48
N11	1518.0 ± 15.60	111.4 ± 9.11	49.44 ± 1.83	1.01 ± 0.06	256.8 ± 4.60	134,871 ± 3004.35
N12	2233.0 ± 7.30	154.20 ± 7.66	64.81 ± 1.62	1.31 ± 0.01	324.3 ± 23.30	138,770 ± 9949.25
N13	1206.0 ± 20.60	91.27 ± 6.65	38.09 ± 0.57	0.86 ± 0.09	249.7 ± 6.30	97,479 ± 2099.55
N14	1841.0 ± 11.70	134.90 ± 0.68	54.10 ± 0.97	1.16 ± 0.06	294.2 ± 16.50	124,773 ± 4617.45
N15	1926.0 ± 45.50	135.40 ± 9.83	53.66 ± 1.00	1.13 ± 0.07	285.5 ± 17.10	130,072 ± 8276.91
N16	2370.0 ± 77.60	171.80 ± 18.22	69.95 ± 10.81	1.63 ± 0.16	556.8 ± 97.40	175,163 ± 11,982.43
N17	1367.0 ± 17.70	95.93 ± 6.74	45.00 ± 1.35	0.98 ± 0.17	241.8 ± 21.60	130,672 ± 3628.27
N18	1447.0 ± 20.80	111.80 ± 5.25	46.35 ± 0.64	1.03 ± 0.05	231.8 ± 4.30	106,926 ± 3488.34
N19	1711.0 ± 31.00	121.70 ± 11.51	53.49 ± 3.04	0.86 ± 0.06	262.3 ± 24.90	133,833 ± 2872.86

Y1, Firmness (g); Y2, Consistency (mJ); Y3, Adhesiveness; Y4, Stringiness (mJ) Y5, Spreadability (g); Y6, Viscosity (cP); The values represent the mean ± standard deviations of three independent measurements.

**Table 5:** Statistical parameters for ANOVA test and quality of fit.

Parameter	Response	R^2^	Q^2^	*p*-Value	Lack of Fit	Model Validity	Reproducibility
Firmness	Y1	0.92	0.82	0.00	0.581	0.86	0.89
Consistency	Y2	0.93	0.83	0.00	0.660	0.89	0.87
Adhesiveness	Y3	0.94	0.86	0.00	0.715	0.92	0.87
Stringiness	Y4	0.96	0.87	0.00	0.706	0.81	0.91
Spreadability	Y5	0.78	0.19	0.006	0.082	0.37	0.96
Viscosity	Y6	0.84	0.45	0.003	0.613	0.88	0.70

R^2^, the fit between the data and the model; Q^2^, the predictive power of the model; Model validity, the validity of the model; Reproducibility, a variation of the response under the identical conditions, compared to the total variation of the response.

**Table 6:** The composition of the optimal formulation and the results of the analysis of optimal formulation.

Composition of the Optimal Formulation				
X1	X2	X3	X4	
2.566	0.066	0.253	20.67	
Results of analysis of optimal formulation				
	Y1	Y2	Y3	Y4
Predicted values	599.61	49.02	25.96	77,662.4
Experimental values	515.3	42.6	24.55	68,365
Difference %	14.06	13.09	5.43	11.97

X1, % Emulsifier; X2, % Co-emulsifier; X3, % Sepigel™305; X4, % Oily phase; Y1, firmness; Y2, consistency; Y3, adhesiveness; Y6, viscosity.

**Table 7:** References used for identification of compounds in the LC/MS method.

Compound	Company Name	Brand Name	Article No.
Caffeic acid	Phytolab GmbH	Phyproof standards	89547
Carnosic acid	Phytolab GmbH	Phyproof standards	89171
Chlorogenic acid	Phytolab GmbH	Phyproof standards	89175
Ferulic acid	Phytolab GmbH	Phyproof standards	89663
Gallic acid	Phytolab GmbH	Phyproof standards	89198
Ellagic acid	Phytolab GmbH	Phyproof standards	89141
Salicylic acid	Merck Group	Sigma-Aldrich	S7401
*trans*-p coumaric acid	Phytolab GmbH	Phyproof standards	89498
Apigenin	Phytolab GmbH	Phyproof standards	89159
Catechin	Phytolab GmbH	Phyproof standards	89172
Chrysin	Merck Group	Supelco	95082
Hyperoside	Phytolab GmbH	Phyproof standards	89227
Kaempferol	Phytolab GmbH	Phyproof standards	89235
Luteolin	Phytolab GmbH	Phyproof standards	89245
Luteolin-*7-O*-glucoside	Phytolab GmbH	Phyproof standards	89724
Myricetin	Phytolab GmbH	Phyproof standards	89252
Naringenin	Phytolab GmbH	Phyproof standards	89738
Pyrocatechol	Phytolab GmbH	Phyproof standards	82372
Quercetin	Phytolab GmbH	Phyproof standards	89262
Quercitrin	Phytolab GmbH	Phyproof standards	89346
Rutoside	Phytolab GmbH	Phyproof standards	89270
Vitexin	Phytolab GmbH	Phyproof standards	89290

**Table 8:** LC/MS parameters of the references.

Name of Standard	Retention Time (min)	*m*/*z* and Main Transition	MRM	Secondary Transitions
Caffeic acid	13.8	179.0 > 135.0	Negative	179.0 > 134.0 179.0 > 89.0
Carnosic acid	32.0	331.2 > 285.1	Negative	
Chlorogenic acid	11.9	353.0 > 191.0	Negative	
Ferulic acid	18.4	193.0 > 134.0	Negative	193.0 > 178.0
Gallic acid	7.0	168.9 > 125.0	Negative	
Ellagic acid	27.2	301.0 > 185.0	Negative	301.0 > 257.0
Salicylic acid	23.5	137.0 > 93.0	Negative	137.0 > 75.0 137.0 > 65.0
trans-*p*-coumaric acid	17.5	163.0 > 119.0	Negative	163.0 > 93.0
Apigenin	28.1	269.0 > 117.0	Negative	
Catechin	10.3	289.0 > 202.9	Negative	
Chrysin	29.7	253.0 > 143.0	Negative	253.0 > 119.0 253.0 > 107.0
Hyperoside	20.3	463.1 > 300.0	Negative	463.1 > 301.0
Kaempferol	27.9	285.0 > 187.0	Negative	285.0 > 151.0 285.0 > 133.0
Luteolin	26.8	287.0 > 153.0	Positive	
Luteolin-*7-O*-glucosid	19.9	447.0 > 284.9	Negative	
Myricetin	13.6	317.0 > 179.0	Negative	317.0 > 151.0 317.0 > 137.0
Naringenin	26.2	271.0 > 119.0	Negative	271.0 > 107.0
Pyrocatechol	11.7	109.0 > 90.6	Negative	109.0 > 52.9
Quercetin	25.4	300.9 > 151.0	Negative	300.9 > 121.0
Quercitrin	22.1	447.0 > 229.9	Negative	
Rutoside	20.2	609.0 > 300.0	Negative	609.0 > 301.0 609.0 > 271.0
Vitexin	18.4	431.0 > 311.0	Negative	

**Table 9:** QTPP and QAs of the developed oil-in-water emulsion.

Cosmetic Dosage Form	Cream	-	Emulsion-Based Product for Topical Delivery of Cosmetic Ingredients
Application site	Topical	-	
Cream design	Oil-in-water emulsion	-	Impact on consumer acceptability
Appearance	Smooth cream	-	Impact on consumer acceptability
Colour	The specific colour of the HM	-	Impact on consumer acceptability; characteristic for the HM content
Odour	Specific for the ingredients; fragrance-free	-	Impact on consumer acceptability; the product is designed for fragilized skin
Physical properties: Texture attributes Firmness-Y1 Consistency-Y2 Adhesiveness-Y3 Stringiness-Y4 Spreadability-Y5 Viscosity-Y6 Content uniformity	500–1000 g 40–100 mJ <30 mJ 0.5–2 mJ <00 g <100,000 cP Homogenous product	Yes Yes Yes - Yes Yes Yes	To ensure the performance of the delivery system and the sensory performance Impact on the sensorial profile of the cream, the handling of the product Impact on the sensorial profile of the cream, influences the residence time on the application site Influences the ease of pick-up from the recipient Impact on consumer acceptability Impact on the sensorial profile of the cream, influences the residence time on the application site Influences the performance of the formulation
Container	Appropriate for the application	-	Influences the stability and safety

**Table 10:** Input variables of the experimental plan.

Independent Variable	Function
X1: Sucrose polystearate & hydrogenated polyisobutene (Emulgade^®^ Sucro)	Oil-in-water emulsifier
X2: Cetearyl glucoside	Co-emulsifier
X3: Polyacrylamide & C13-14 Isoparaffin & Laureth-7 (Sepigel™305)	Thickening and texturizing agent, stabilizer
X4: Cetylstearyl alcohol, caprylic/capric triglycerides, coco-caprylate, avocado oil, almond oil, argan oil	Oily phase of the oil-in-water emulsion

**Table 11:** Experimental design matrix.

Exp Name	X1	X2	X3	X4
N1	2.5	0	0.2	20
N2	3.5	0	0.2	20
N3	2.5	1	0.2	20
N4	3.5	1	0.2	20
N5	2.5	0	0.4	20
N6	3.5	0	0.4	20
N7	2.5	1	0.4	20
N8	3.5	1	0.4	20
N9	2.5	0	0.2	30
N10	3.5	0	0.2	30
N11	2.5	1	0.2	30
N12	3.5	1	0.2	30
N13	2.5	0	0.4	30
N14	3.5	0	0.4	30
N15	2.5	1	0.4	30
N16	3.5	1	0.4	30
N17	3	0.5	0.3	25
N18	3	0.5	0.3	25
N19	3	0.5	0.3	25

X1—emulsifier ratio, X2—co-emulsifier ratio, X3—thickening agent ratio, and X4—oily phase ratio.

## Data Availability

Not applicable.

## Supplementary Materials

The following supporting information can be downloaded at: https://www.mdpi.com/article/10.3390/plants12020248/s1, Figures S1–S22: HPLC chromatograms and mass spectra of compounds analyzed from the HM; Figure S23: Ishikawa diagram, Figure S24: Response surface plots; Table S1: FMEA risk assessment, Table S2: LC/MS mobile-phase gradient composition.
