# Supplementary material for: Chemical Profile and Biological Effects of an Herbal Mixture for the Development of an Oil-in-Water Cream
Source: Plants (Basel). 2023 Jan 5;12(2):248. doi: 10.3390/plants12020248 (PMC9861053; doi:10.3390/plants12020248)
Supplement: Supplementary file 1 [file plants-12-00248-s001.zip › plants-2031410-supplementary.pdf]

Supplementary material

Chemical Profile and Biological Effects of an Herbal Mixture for the Development of an Oil-in-Water Cream

Diana Antonia Safta [1,†], Irina Ielciu [2,†], Raffaela Șuștic [3], Daniela Hanganu [4,*], Mihaela Niculae [5], Mihai Cenariu [5], Emoke Pall [5], Mirela Liliana Moldovan [1], Marcela Achim [3], Cătălina Bogdan [1,†] and Ioan Tomuță [3,†]

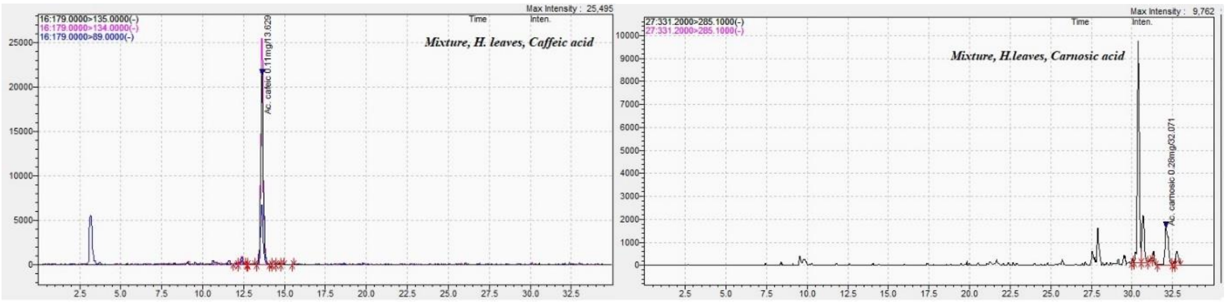

Figure S1. HPLC-MS Chromatograms of caffeic and carnosic acids from HM

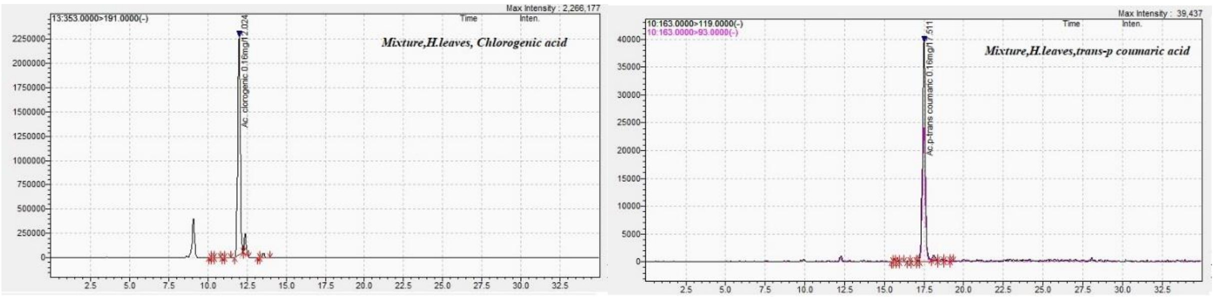

Figure S2. HPLC-MS Chromatograms of chlorogenic and p-coumaric acids from HM

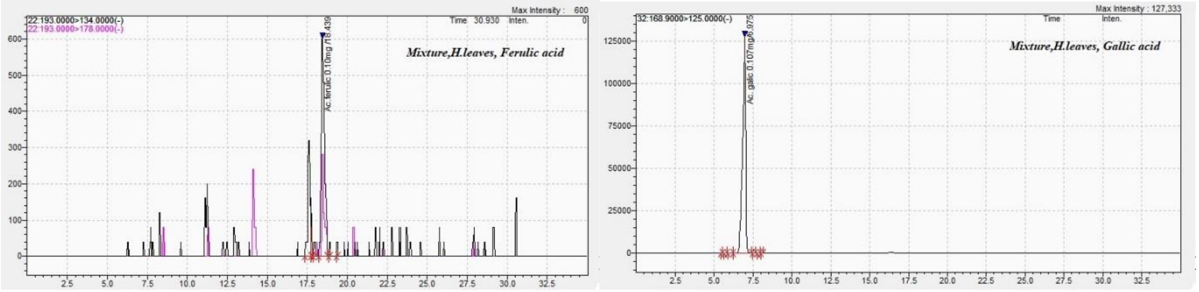

Figure S3. HPLC-MS Chromatograms of ferulic and gallic acids from HM

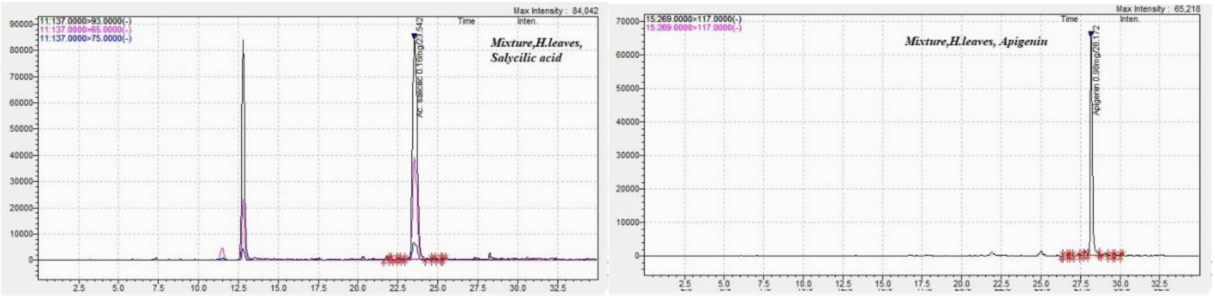

Figure S4. HPLC-MS Chromatograms of salycilic acid and apigenin from HM

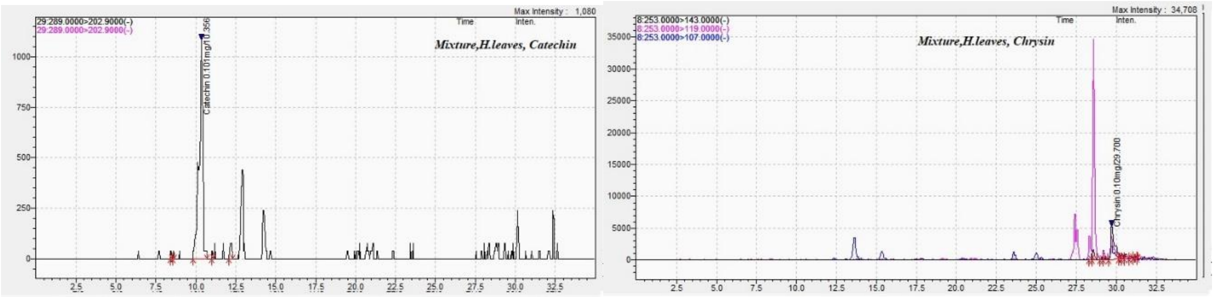

Figure S5. HPLC-MS Chromatograms of catechin and chrysin from HM

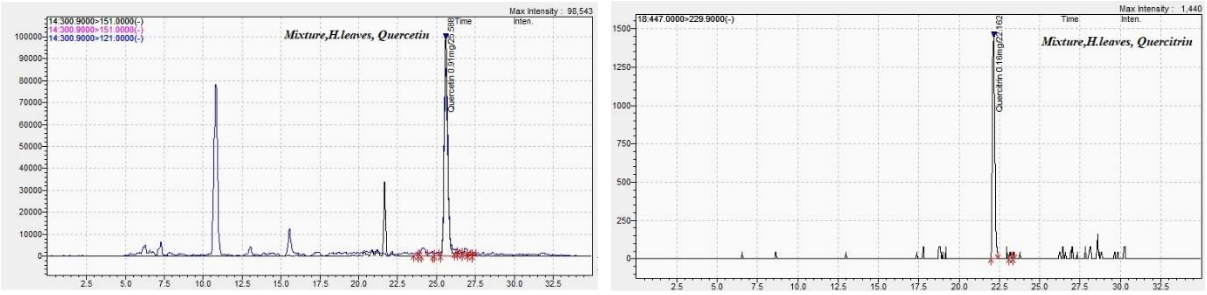

Figure S6. HPLC-MS Chromatograms of quercetin and quercitrin from HM

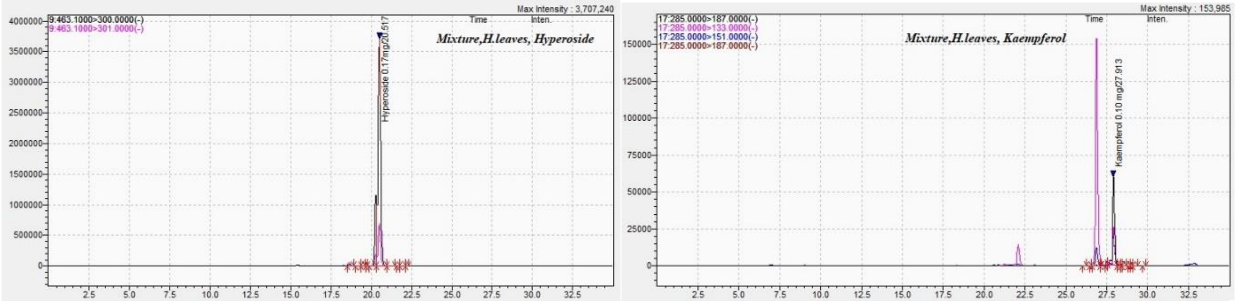

Figure S7. HPLC-MS Chromatograms of hyperoside and kaempferol from HM

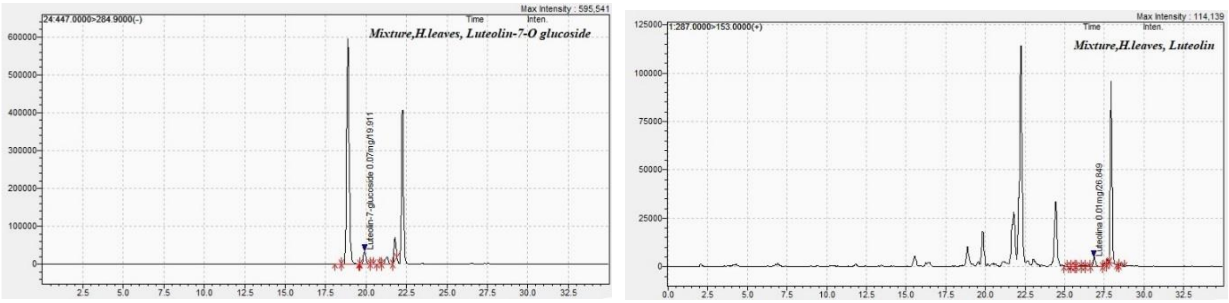

Figure S8. HPLC-MS Chromatograms of luteolin-7-O-glucoside and luteolin from HM

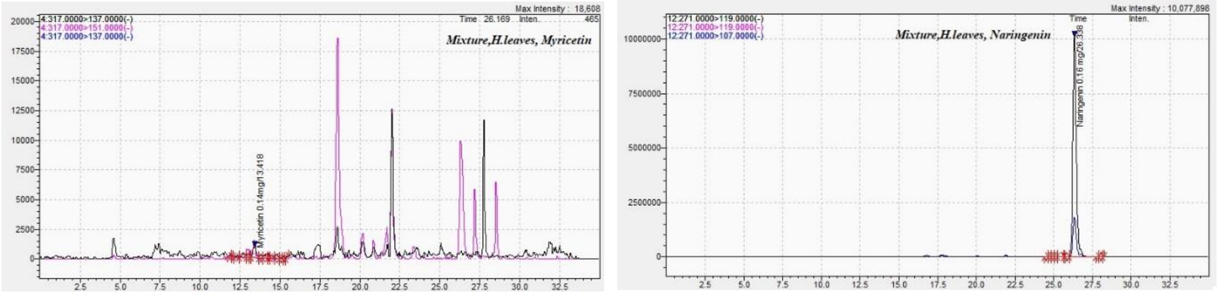

Figure S9. HPLC-MS Chromatograms of myricetin and naringenin from HM

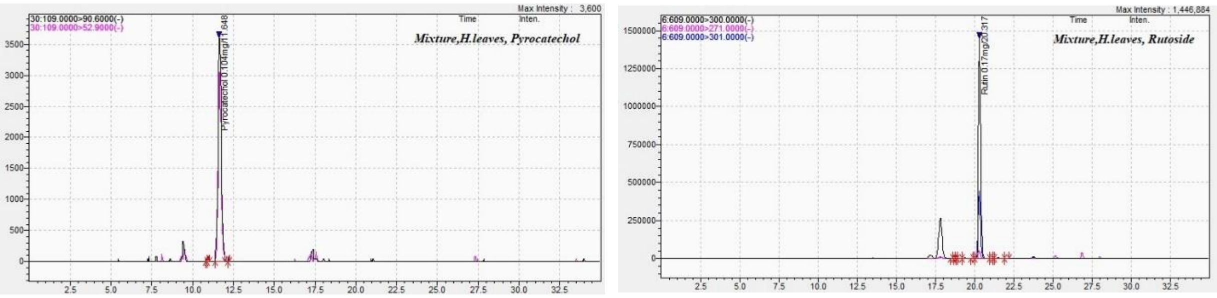

Figure S10. HPLC-MS Chromatograms of pyrocatechol and rutoside from HM

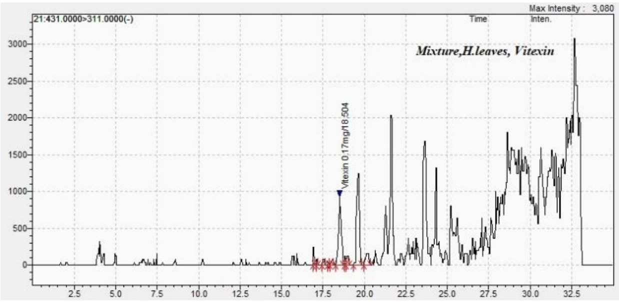

Figure S11. HPLC-MS Chromatogram of vitexin from HM

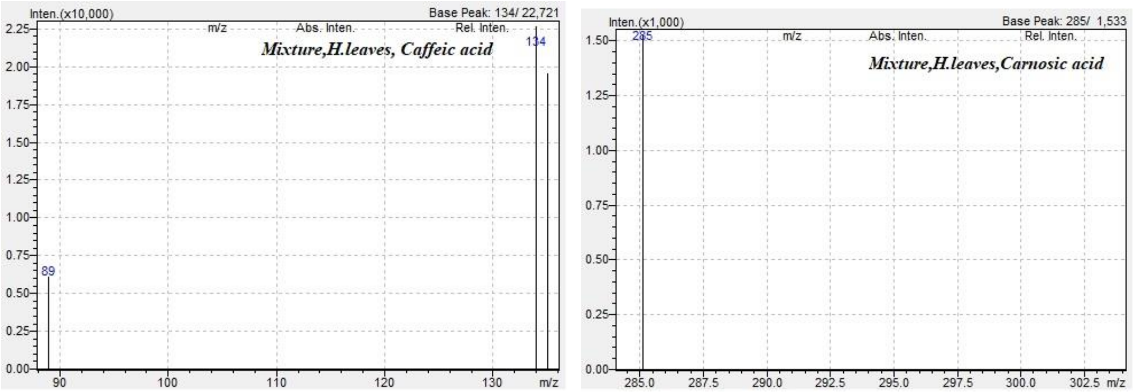

Figure S12. The mass spectra of the HPLC-MS analyzed caffeic and carnosic acids from HM

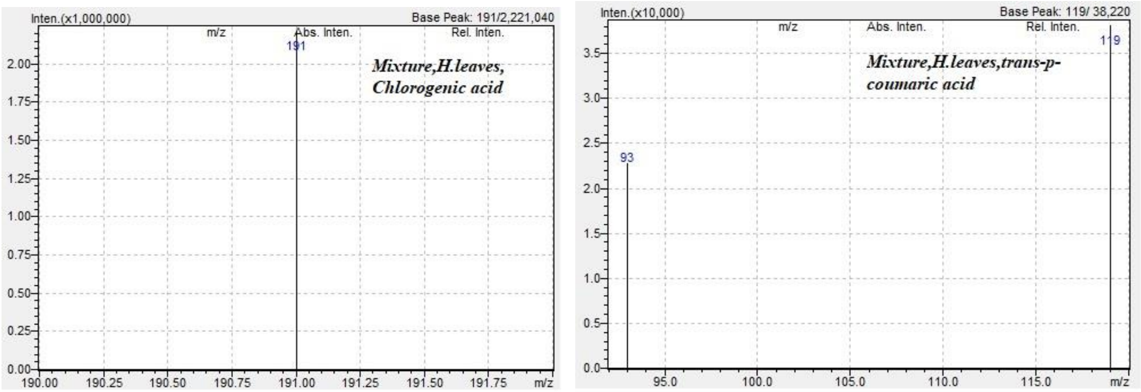

Figure S13. The mass spectra of the HPLC-MS analyzed chlorogenic and p-coumaric acids from HM

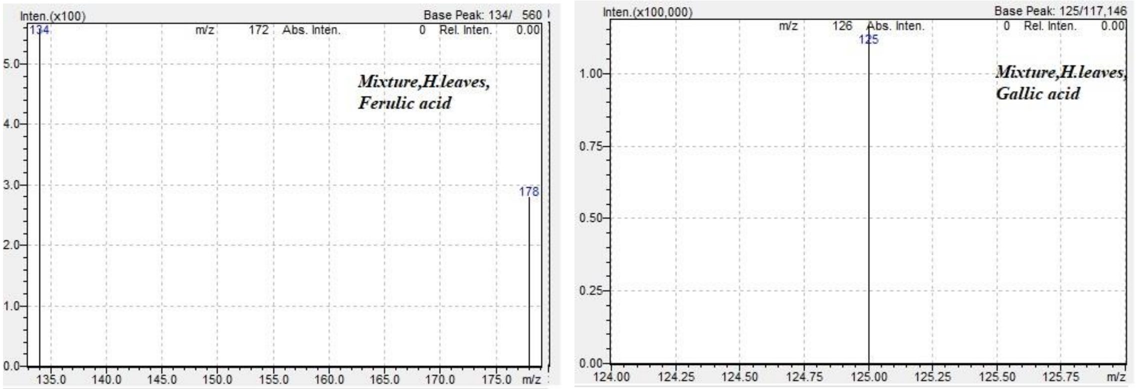

Figure S14. The mass spectra of the HPLC-MS analyzed ferulic and gallic acids from HM

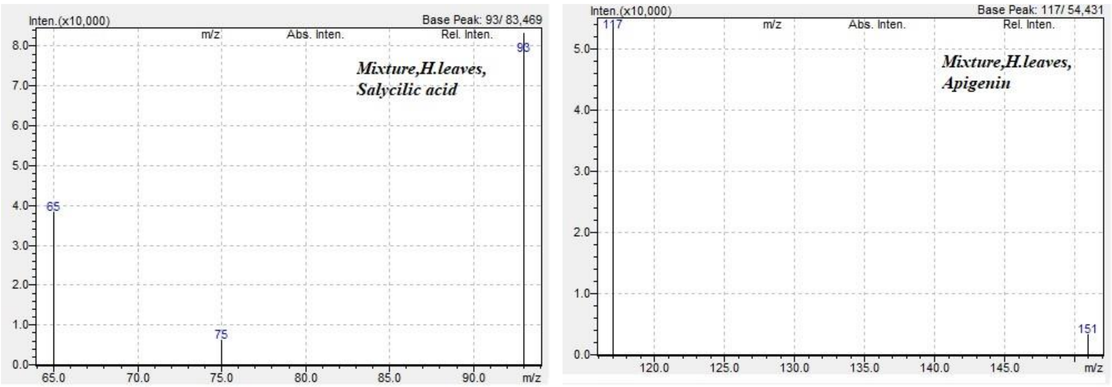

Figure S15. The mass spectra of the HPLC-MS analyzed salycilic acid and apigenin from HM

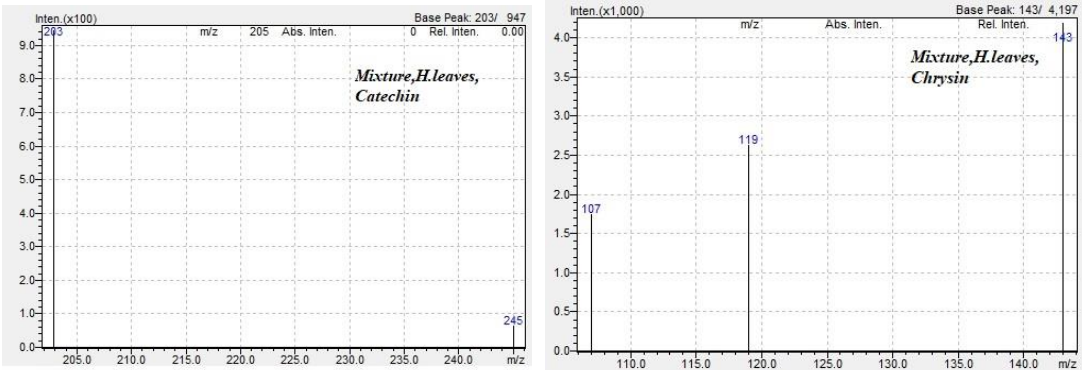

Figure S16. The mass spectra of the HPLC-MS analyzed catechin and chrysin from HM

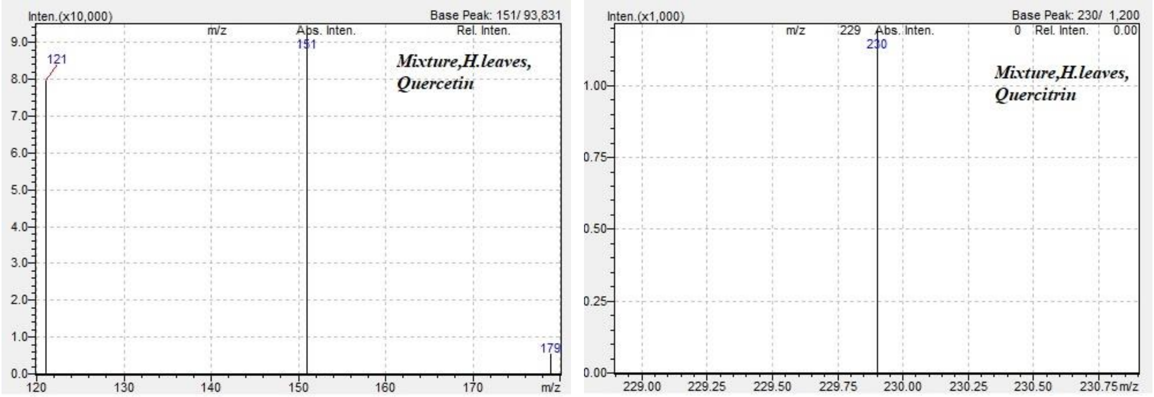

Figure S17. The mass spectra of the HPLC-MS analyzed quercetin and quercitrin from HM

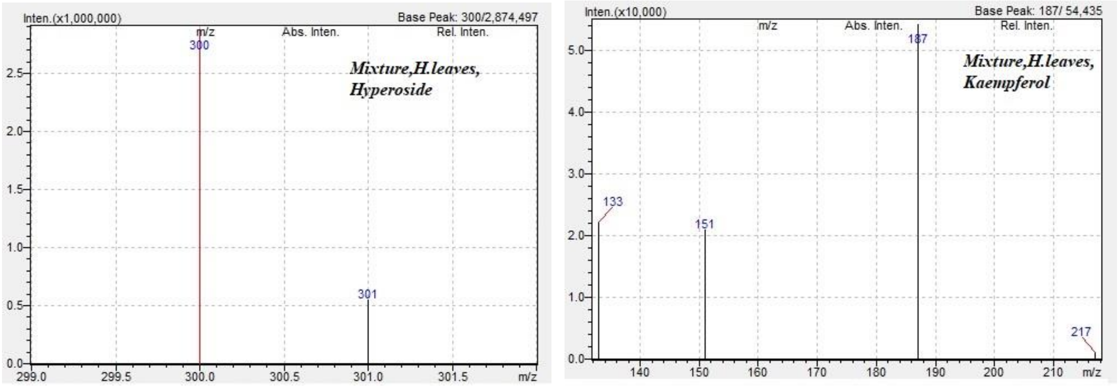

Figure S18. The mass spectra of the HPLC-MS analyzed hyperoside and kaempferol from HM

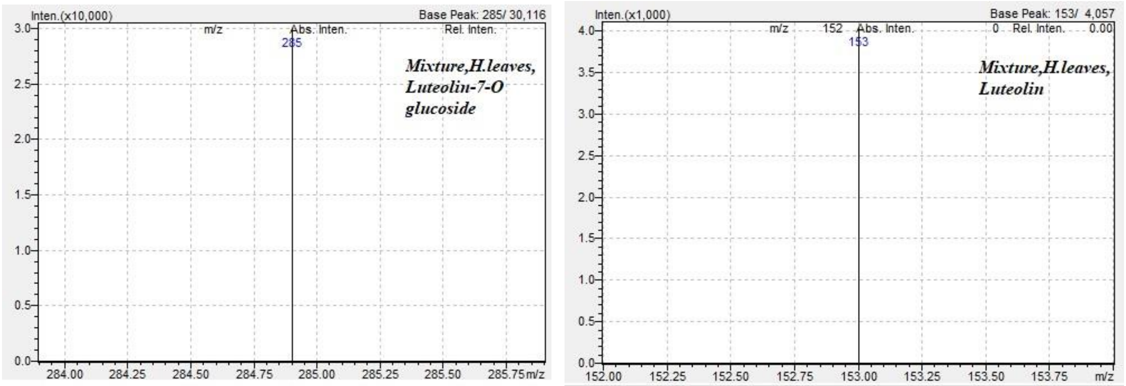

Figure S19. The mass spectra of the HPLC-MS analyzed luteolin-7-O-glucoside and luteolin from HM

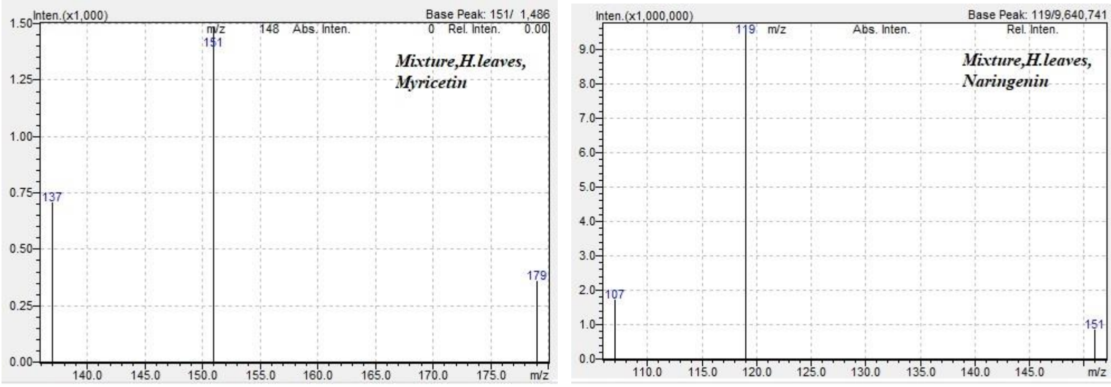

Figure S20. The mass spectra of the HPLC-MS analyzed myricetin and naringenin from HM

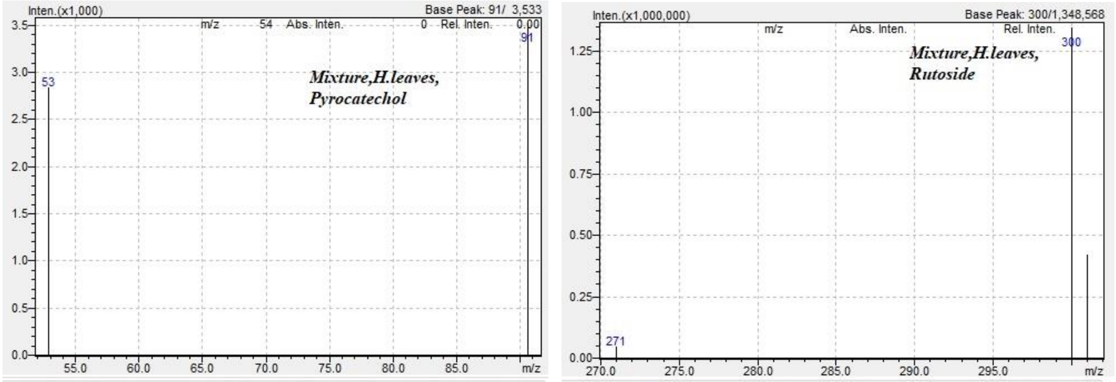

Figure S21. The mass spectra of the HPLC-MS analyzed pyrocatechol and rutoside from HM

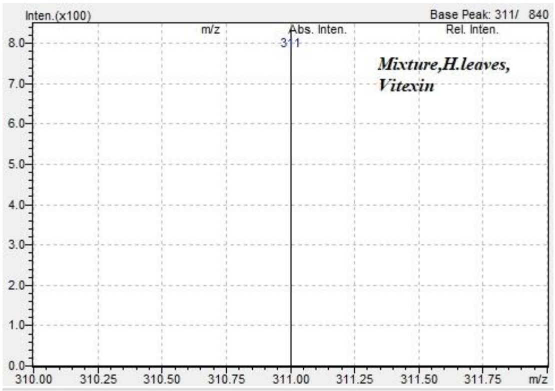

Figure S22. The mass spectra of the HPLC-MS analyzed vitexin from HM

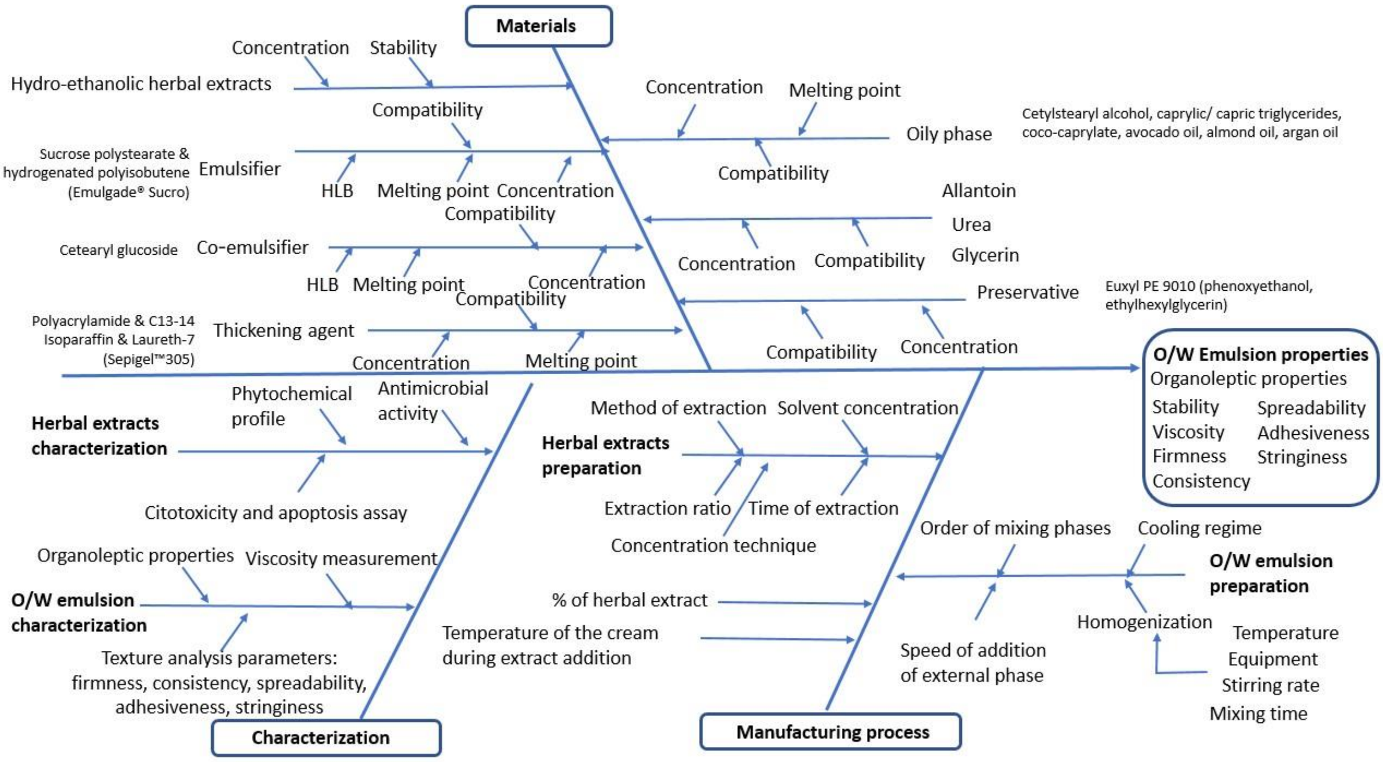

Figure S23. Ishikawa diagram

*HLB-hydrophilic-lipophilic balance, O/W- oil-in-water

Table S1. FMEA risk assessment

CPPss		Potential failure mode	Potential failure effects	O	S	D	RPN
Critical formulation factors							
Hydro-ethanolic herbal extracts	Concentration	Improper concentration	Efficiency	1	4	5	20
	Stability	Alteration of phyto-compounds	Efficiency	3	3	5	45
	Compatibility	Incompatibility with other ingredients	Stability, organoleptic properties, viscosity, texture parameters	3	5	3	45
Emulsifier	HLB	Failure of emulsification process	Stability, organoleptic properties	5	5	2	50

	CPPss	Potential failure mode	Potential failure effects	O	S	D	RPN
	Melting point	Great difference melting point comparative to other ingredients	Viscosity, texture parameters	4	4	1	16
	Concentration	Inappropriate concentrations	Viscosity, texture parameters	5	5	3	75
	Compatibility	Incompatibility with other ingredients	Stability, organoleptic properties, viscosity, texture parameters	3	5	3	45
Co-emulsifier	HLB	Incapacity of emulsification	Stability, organoleptic properties	5	5	2	50
	Melting point	Great difference melting point comparative to other ingredients	Stability, viscosity, texture parameters	4	4	1	16
	Concentration	Improper concentration	Viscosity, texture parameters	5	5	3	75
	Compatibility	Incompatibility with other ingredients	Stability, organoleptic properties, viscosity, texture parameters	3	5	3	45
Thickening agent	Melting point	Great difference melting point comparative to other ingredients	Stability, viscosity, texture parameters	4	4	1	16
	Concentration	Inappropriate concentrations	Viscosity, texture parameters	5	5	3	75
	Compatibility	Incompatibility with other ingredients	Stability, organoleptic properties, viscosity, texture parameters	3	5	3	45
Oily phase	Melting point	Great difference melting point comparative to other ingredients	Stability, viscosity, texture parameters	4	4	1	16
	Concentration	Inappropriate concentrations	Viscosity, texture parameters	5	5	3	75
	Compatibility	Incompatibility with other ingredients	Stability, organoleptic properties, viscosity, texture parameters	3	5	3	45
Allantoin, urea, glycerine	Concentration	Inappropriate concentrations	Efficiency	2	3	3	18
	Compatibility	Incompatibility with other ingredients	Stability, organoleptic properties	4	3	1	12
Preservative	Concentration	Inappropriate concentrations	Stability	3	3	3	27
	Compatibility	Incompatibility with other ingredients	Stability, organoleptic properties	3	3	1	9
		Critical process parameters					
Herbal extracts preparation	Method of extraction	Improper extraction	Efficiency	3	3	5	45
	Solvent concentration	Improper extraction	Efficiency	3	3	5	45
	Extraction ratio	Improper extrac-	Efficiency	3	3	5	45

CPPss		Potential failure mode	Potential failure effects	O	S	D	RPN
		tion					
	Time of extraction	Improper extraction	Efficiency	3	3	5	45
	Concentration technique	Improper concentration of extracts	Efficiency	3	3	5	45
% of herbal extract		Improper efficiency	Efficiency	3	3	5	45
Temperature of the cream during extract addition		Inappropriate temperature	Stability, organoleptic properties, viscosity, texture parameters, stability	4	4	2	32
Emulsion preparation	Order of mixing phases	Improper order	Stability, organoleptic properties, viscosity, texture parameters, stability	4	4	2	32
	Cooling regime	Inappropriate temperature	Stability, organoleptic properties, viscosity, texture parameters, stability	4	4	2	32
	Speed of addition of external phase	Improper speed	Stability, organoleptic properties, viscosity, texture parameters, stability	4	4	2	32
	Homogenization- temperature	Inappropriate temperature	Stability, organoleptic properties, viscosity, texture parameters, stability	4	4	2	32
	Homogenization-equipment	Improper equipment	Stability, organoleptic properties, viscosity, texture parameters, stability	4	4	2	32
	Stirring rate	Improper stirring rate	Stability, organoleptic properties, viscosity, texture parameters, stability	4	4	2	32
	Mixing time	Inappropriate time	Stability, organoleptic properties, viscosity, texture parameters, stability	4	4	2	32

CPPs – Critical Process Parameters; O – occurrence; S – severity; D – detectability; RPN – risk priority number;

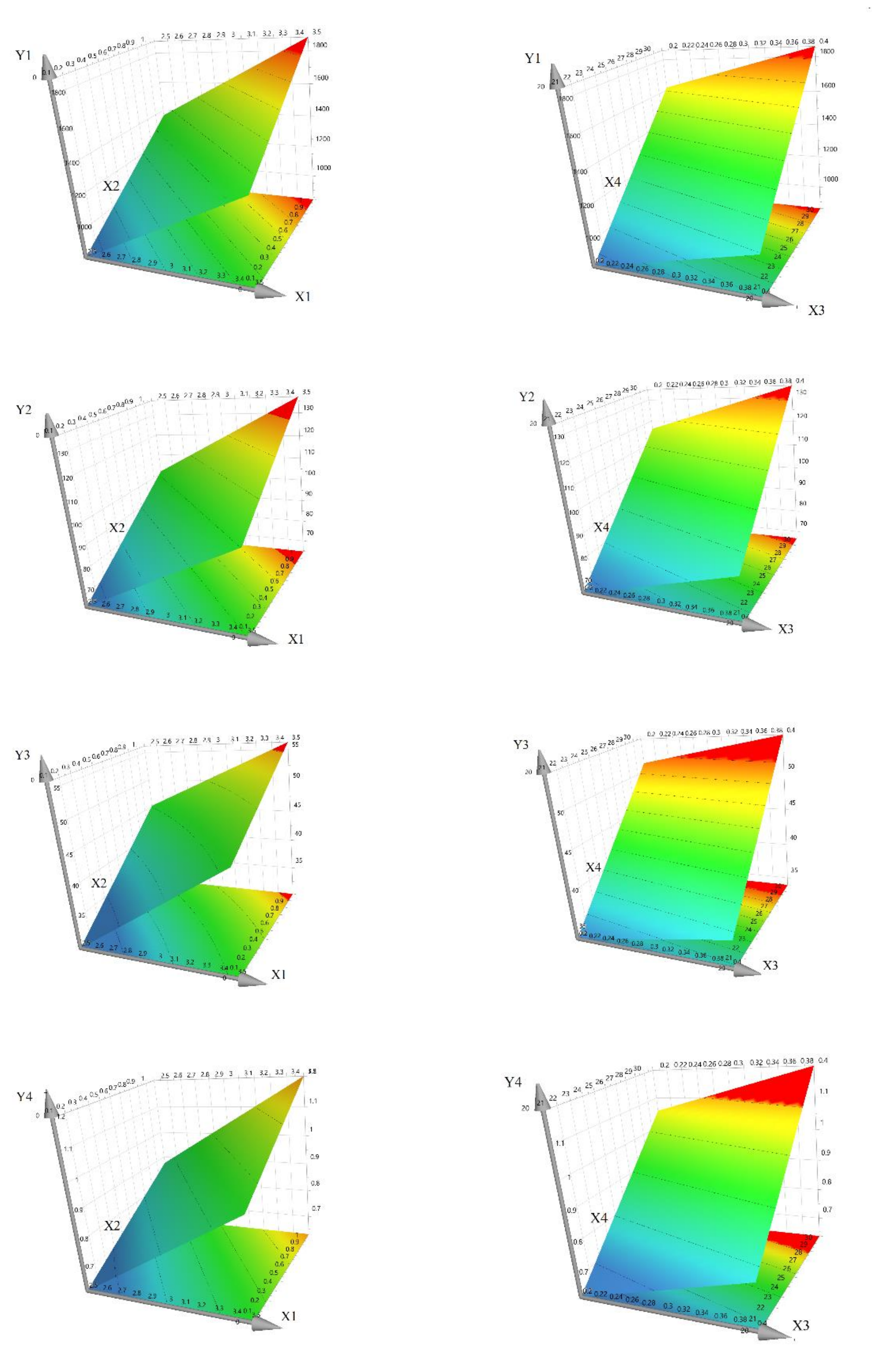

Figure S24. Response surface plots (X1- emulsifier ratio, X2- co-emulsifier ratio, X3 -thickening agent ratio, and X4 - oily phase ratio; Y1 - firmness; Y2 – consistency; Y3 - adhesiveness; Y4 -stringiness; Y5 - spreadability; Y6- viscosity)

Table S2. LC/MS mobile-phase gradient composition

Time, min	Methanol	Water	2 % formic acid in water
0.00	5	90	5
3.00	15	70	15
6.00	15	70	15

9.00	21	58	21
13.00	21	58	21
18.00	30	41	29
22.00	30	41	29
26.00	50	0	50
29.00	50	0	50
29.01	5	90	5
35.00	5	90	5
